# Transcriptional inhibition by CDK7/9 inhibitor SNS-032 abrogates oncogene addiction and reduces liver metastasis in uveal melanoma

**DOI:** 10.1186/s12943-019-1070-7

**Published:** 2019-09-16

**Authors:** Jing Zhang, Shenglan Liu, Qianyun Ye, Jingxuan Pan

**Affiliations:** State Key Laboratory of Ophthalmology, Zhongshan Ophthalmic Center, Sun Yat-sen University, 54 South Xianlie Road, Guangzhou, 510060 People’s Republic of China

**Keywords:** Uveal melanoma, SNS-032, Transcription, Apoptosis, Cancer stem-like cells, Self-renewal, KLF4, Cell motility, RhoA, Liver metastasis

## Abstract

**Background:**

Life of patients with uveal melanoma (UM) is largely threatened by liver metastasis. Little is known about the drivers of liver organotropic metastasis in UM. The elevated activity of transcription of oncogenes is presumably to drive aspects of tumors. We hypothesized that inhibition of transcription by cyclin-dependent kinase 7/9 (CDK7/9) inhibitor SNS-032 diminished liver metastasis by abrogating the putative oncogenes in charge of colonization, stemness, cell motility of UM cells in host liver microenvironment.

**Methods:**

The effects of SNS-032 on the expression of the relevant oncogenes were examined by qRT-PCR and Western blotting analysis. Proliferative activity, frequency of CSCs and liver metastasis were evaluated by using NOD-SCID mouse xenograft model and NOG mouse model, respectively.

**Results:**

The results showed that CDK7/9 were highly expressed in UM cells, and SNS-032 significantly suppressed the cellular proliferation, induced apoptosis, and inhibited the outgrowth of xenografted UM cells and PDX tumors in NOD-SCID mice, repressed the cancer stem-like cell (CSC) properties through transcriptional inhibition of stemness-related protein Krüppel-like factor 4 (KLF4), inhibited the invasive phonotypes of UM cells through matrix metalloproteinase 9 (MMP9). Mechanistically, SNS-032 repressed the c-Myc-dependent transcription of *RhoA* gene, and thereby lowered the RhoA GTPase activity and actin polymerization, and subsequently inhibited cell motility and liver metastasis.

**Conclusions:**

In conclusion, we validate a set of transcription factors which confer metastatic traits (e.g., KLF4 for CSCs, c-Myc for cell motility) in UM cells. Our results identify SNS-032 as a promising therapeutic agent, and warrant a clinical trial in patients with metastatic UM.

## Introduction

Uveal melanoma (UM) is the most common intraocular malignancy in adults which arises from melanocytes of the choroid, ciliary and iris. It accounts for 5% of all melanomas [1]. Although the primary tumors can be successfully treated with enucleation or radiotherapy, distal metastasis, predominantly (~ 90%) to the only organ liver is observed in half of the UM patients [2]. The prognosis of the patients with metastatic UM is poor with median overall survival of 2 to 8 months [3].

UM is biologically and clinically different from cutaneous melanoma which commonly harbors the activating mutations in BRAF or NRAS [4]. Clinical trials demonstrated that patients with even advanced cutaneous melanoma responded well to immune checkpoint inhibitors such as ipilimumab and pembrolizumab, the monoclonal antibody that blocks the cytotoxic T-lymphocyte-associated antigen 4 (CTLA-4) receptor and programmed cell death-1 (PD-1) receptor, respectively, while patients with advanced UM did not all [5]. Actually, no FDA-approved standard therapy is available for metastatic UM currently. Therefore, novel effective therapeutic drugs are urgently needed for the treatment of patients with metastatic UM.

The past decade witnessed the identification of GNAQ/GNA11-Yes-associated protein (YAP) cascade, a profound progress in the pathogenesis of UM. Whole-genome sequencing [6] has demonstrated that mutually exclusive gain of function mutations in GNAQ (Gα_q_) or GNA11 (Gα_11_), which encodes the α subunit of heterotrimeric G protein, are found in ~ 80% of patients with UM. Gain-of-function mutations in GNAQ (Gα_q_) or GNA11 (Gα_11_), leading to activation of the protein kinase C (PKC) and mitogen-activated protein kinase (MAPK) pathway via phospholipase C-β (PLCβ), are presumably believed to be drive tumorigenecity of UM [7]. Combinational treatment with PKC and MEK inhibitors showed modest effective in UM cells with Gαq/11 mutations [8]. Unfortunately, no clinical benefit was observed in phase III clinical trial with selumetinib (AZD6244), a selective, non-ATP competitive inhibitor of MEK1/2 in patients with metastatic UM [9]. Recent studies showed that activation of the transcriptional cofactor YAP by mutated GNAQ or GNA11 through Trio-Rho/Rac signaling was required for this mutation-induced tumorigenesis in UM [10, 11]. The YAP-inhibitory drug Verteporfin selectively suppresses tumorigenesis in UM with Gαq/11 mutations [10–12]. However, the clinical efficacy of Verteporfin in patients with metastatic UM remains to be evaluated.

In contrast to improvement in understanding the drivers of the pathogenesis of UM, little is known about the drivers of expansion of colonized tumor cells in liver in patients with UM. Highly metastatic UM patients are characterized by monosomy of chromosome 3 and gain of 8q [5]. Recurrent somatic mutations in BRCA1-associated protein 1 (BAP1), splicing factor 3B subunit 1 (SF3B1), and eukaryotic translation initiation factor 1A (EIF1AX) are found in metastatic UM. Inactivating somatic mutations in the *BAP1* gene were identified in 80% of metastasizing UM, making BAP1 pathway a potentially valuable therapeutic target [13]. Recently, the preclinical mouse model generated by melanocyte-specific expression of GNA11^Q209L^ with or without homozygous BAP1 loss recapitulates features of aggressive UM suggesting the fundamental role of GNA11 in the initiation of UM [7]. The function of SF3B1 and EIF1AX is not known in metastatic UM. In addition, the receptor tyrosine kinase c-Met is highly expressed in patients with metastatic UM. Crizotinib has been shown to inhibit the phosphorylation of c-Met and reduce the development of metastasis in a mouse model of metastatic UM [14]. No results of clinical trial of c-Met inhibitors have been reported. Taken together, no clear and precise drivers of metastasis in UM have been identified so far.

Certain tumor shows extraordinarily dependence on continuous expression of oncogenes. Oncogene addiction involved in tumor initiation and progression is the molecular basis for targeted therapy in cancer [15]. This anti-tumor rationale of abrogating oncogene addiction by targeting transcription is of particular importance for those tumors in which no exact drivers have been identified [16]. For example, targeting bromodomain 4 (BRD4) with JQ1 potently inhibits the growth and metastasis in certain cancers which addiction to the transcription [17]. The cyclin-dependent kinases (CDKs) play important role in regulation of cell-cycle progression and transcription. CDK7 and CDK9, two transcriptional CDKs, promote the initiation and elongation of transcription, respectively. As a key component of transcription factor II H (TFIIH), CDK7 facilitates transcription initiation by phosphorylating serines 5 and 7 (Ser5 and 7) in the carboxy terminal domain (CTD) of RNA polymerase II (RNA Pol II). CDK9, as part of positive transcription elongation factor-b (P-TEFb), is required for elongation by phosphorylating Ser2 in CTD of RNA Pol II [18]. SNS-032 (BMS-387032) is a potent and selective CDK7/9 inhibitor. Studies from us and others have indicated that SNS-032 has promising antitumor activities in leukemia and solid tumors [19, 20]. A phase I study in patients with metastatic refractory solid tumors demonstrated that SNS-032 was well tolerated and may be feasible for oral administration [21]. SNS-032 showed modest clinical activity in patients with advanced chronic lymphocytic leukemia and multiple myeloma in phase I clinical trial [22].

Although growth in the primary tumors and the metastatic tumors may be determined by different specific oncogenes, we propose that expansion of colonized tumor cells in the second sites during metastasis may be also attributed to oncogene addiction in which targeted therapy may be developed. In this study, we hypothesized that inhibition of transcription by CDK7/9 inhibitor diminished liver metastasis by abrogating the putative oncogenes in charge of growth, colonization, stemness, cell motility of UM cells in the liver microenvironment. Our results demonstrated that SNS-032 potently inhibited cellular proliferation, induced apoptosis, reduced migration and invasion, eliminated CSCs of UM, and ultimately inhibited liver metastasis in UM. These findings idenfity SNS-032 a promising agent against UM cells, and warrant a clinical trial for patients with hepatic metastasis of UM.

## Materials and methods

### Cell culture

Human UM cell lines 92.1, Mel270, Omm1 and Omm2.3 were cultured in RPMI 1640 medium (Thermo Fisher Scientific, Shanghai, China) supplemented with 10% fetal bovine serum (FBS) (Hyclone, Guangzhou, China), 2 mM L-glutamine, 100 units/ml penicillin, and 100 μg/ml streptomycin. Human adult retinal pigmented epithelium (ARPE-19) cells purchased from the American Type Culture Collection (ATCC, Manassas, VA) were culture in DMEM/F12 medium supplemented with 10% FBS [23]. Human embryonic kidney 293 T cells and MP41 cells obtained from ATCC were cultured in DMEM supplemented with 10% FBS or RPMI1640 supplemented with 20% FBS, respectively. Cells were kept at 37 °C in a humidified incubator with 5% CO_2._ All the cell lines were tested and authenticated by using short tandem repeat (STR) matching analysis every 6 months and confirmed to be no mycoplasma contamination.

### Reagents and DNA constructs, Western blot analysis, cell viability assay, colony-formation assay, apoptosis assay by flow cytometry, measurement of mitochondrial transmembrane potential, real-time quantitative RT-PCR (qRT-PCR), dual luciferase reporter assay, melanosphere-formation assay, aldehyde dehydrogenase positive cells assay, limiting dilution assay in NOD-SCID mice, wound-healing scratch assay, migration and invasion assay, lentivirus transduction, F-actin staining assay, small GTPases activities assay, chromatin immunoprecipitation assay

All above methods were performed as described previously reported [23–28], with details provided in the Additional file 2. The primers for qRT-PCR and ChIP assay are listed in Additional file 1: Tables S1 and S2, respectively.

### Tumor xenograft experiments

Omm1 cells (3 × 10^6^ in 200 μl PBS) subcutaneously inoculated into the flanks of the male NOD-SCID mice (4 to 6-week-old) purchased from Vital River Laboratory Animal Technology Co., Ltd. (Beijing, China) and bred at the animal facility of Sun Yat-sen University. Tumors were measured every other day with calipers and calculated using the following formula: *a*^2^ × *b* × 0.4, where *a* is the smallest diameter and *b* is the diameter perpendicular to a. Four weeks later, when the tumors reached to ~ 100 mm^3^, the mice were randomly divided into two groups (*n* = 8 per group) and received treatment with vehicle (PBS with DMSO<0.1%) or SNS-032 (15 mg/kg/day, i.p.) on a three-days-on/two-days-off schedule for 14 days. Animals were then euthanized, and tumor xenografts were removed, photographed, and weighed. Tumor tissues were fixed with formalin for immunohistochemical (IHC) and haematoxylin and eosin (H&E) staining or prepared cell lysates for Western blot analysis [19, 23].

### Patient-derived xenograft model

The in vivo anti-UM effects of SNS-032 were evaluated by using the widely accepted MP41 patient-derived xenograft (PDX) model [29–32]. In brief, MP41 cells (1 × 10^7^ in 200 μl PBS) subcutaneously inoculated into the flanks of the male NOD-SCID mice. The tumors were removed and cut into small pieces with a volume of 30–60 mm^3^ when grown to ~ 800 mm^3^, and subcutaneously inoculated into the flanks of the NOD-SCID mice. The tumor xenografts were used for experiments after three serial passages. Tumor pieces of ~ 60 mm^3^ were subcutaneously grafted into the flanks of the NOD-SCID mice. When tumors grown to ~ 200 mm^3^, the mice were randomly divided into vehicle or SNS-032 groups (*n* = 8 per group) for 30 days treatment according to the same compound administration scheme as aforementioned. The mice were sacrificed when the tumor volume in vehicle group grown to ~ 1000 mm^3^.

### Liver metastasis mouse model

Mel270- or Omm2.3-luciferase cells (5 × 10^5^ per mouse in 50 μl PBS) were intrasplenically injected into the NOG mice (Vital River, Beijing, China). Twenty-four hours later, the mice were randomly divided into two groups (*n* = 5 for Mel270-luc cells or *n* = 3 for Omm2.3-luc cells, respectively) and received treatment with vehicle (PBS with DMSO<0.1%) or SNS-032 (15 mg/kg/day, i.p.) on a three-days-on/two-days-off schedule for 28 days. The mice were monitored by in vivo bioluminescence using the Xenogen VivoVision IVIS 100 system (PerkinElmer) after i.p. injection of D-luciferin at a dose of 150 mg/kg. The mice were then euthanized, the livers were removed and fixed in Bouin’s solution (75% saturated picric acid, 25% formaldehyde, and 5.0% acetic acid) overnight. The nodules on hepatic surface were counted. The liver sections were performed H&E staining [23].

All the animal experiments were approved by the Sun Yat-sen University Institutional Animal Care and Use Committee.

### IHC and H&E staining assay

Tissue sections (4-μm) from formalin-fixed, paraffin-embedded tumor xenografts were immunostained with anti-Ki67 and –active caspase-3 antibodies (1:100 dilution) using the MaxVision kit (Maixin, Fuzhou, China) according to the standard protocol as described [23, 28]. Color was developed with 0.05% diaminobenzidine and the sections were counterstained with hematoxylin. The sections were mounted with Permount™ Mounting Medium and photographed under an inverted fluorescence microscope.

### Statistical analysis

All experiments were performed 3 times, and results are reported as mean ± standard deviation (SD), unless otherwise stated. GraphPad Prism 5.0 Software (San Diego, CA) was used for statistical analysis. Comparisons between 2 groups were analyzed by Student’s *t* test and comparisons of multiple groups by one-way ANOVA with post hoc intergroup comparison with Tukey’s test. A *P* < 0.05 was considered statistically significant.

## Results

### SNS-032 inhibits proliferation of UM cells through blocking YAP signaling

**Fig. 1:**
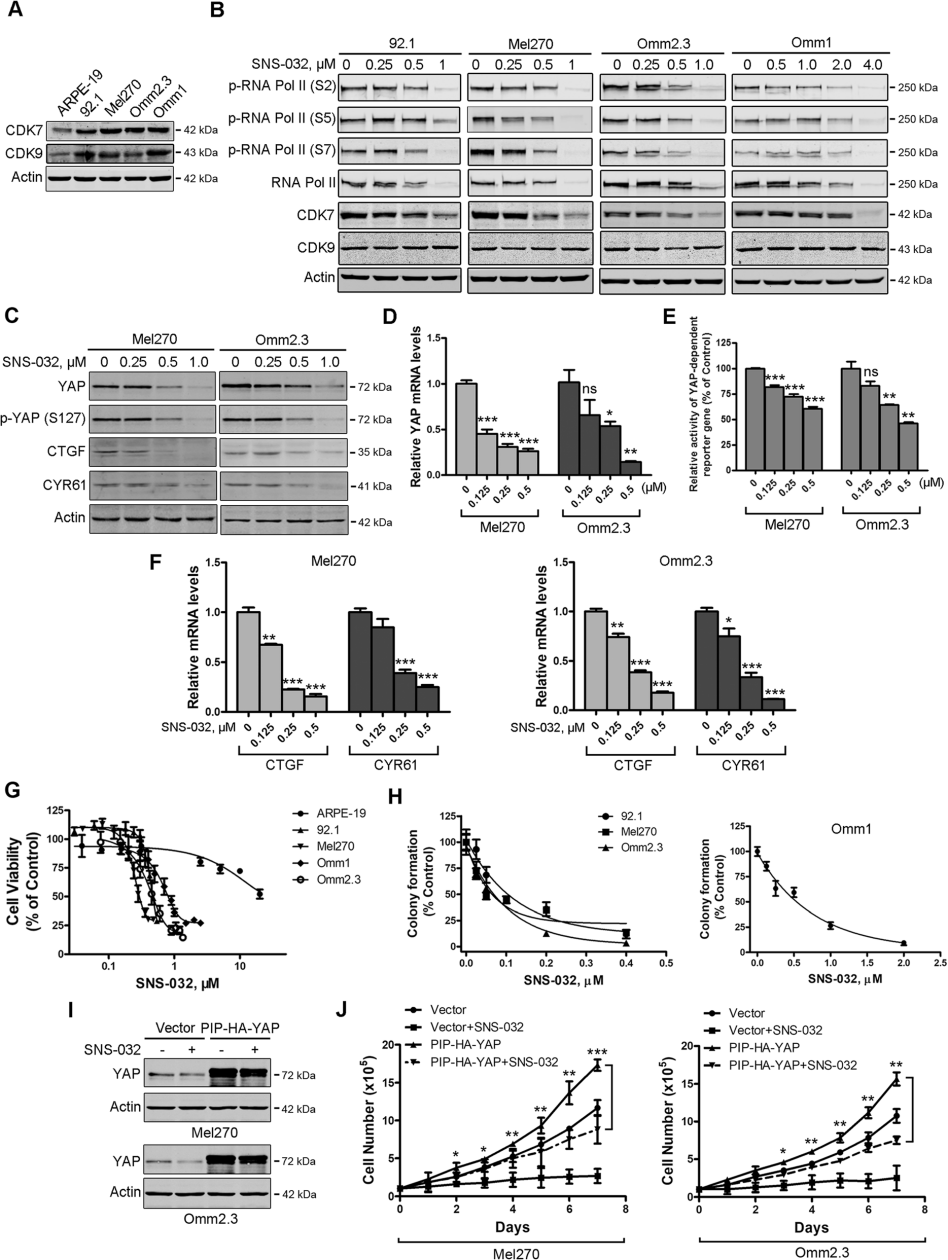
SNS-032 inhibits cellular growth through blocking YAP signaling in uveal melanoma (UM) cells. **a** The protein levels of CDK7/9 in UM cells and ARPE-19 cells were examined by Western blot analysis. **b** SNS-032 inhibited CTD phosphorylation of RNA Pol II in UM cells. Cells were treated with increasing concentration of SNS-032 for 48 h, Western blot analysis with the indicated antibodies was performed. **c** SNS-032 blocked YAP signaling in UM cells. Mel270 and Omm2.3 cells were treated with SNS-032 for 48 h, the protein levels of YAP, phospho-YAP (S127) and its downstream targets CTGF and CYR61 were examined by Western blot analysis. **d** SNS-032 inhibited gene transcription of *YAP*. The UM cells were treated with SNS-032 for 24 h, the mRNA levels of *YAP* were detected by qRT-PCR analysis. **e** SNS-032 suppressed YAP-mediated gene transcription. UM cells were co-transfected with Gal4BD-TEAD4, G5-luciferase reporter (contains five Gal4-binding sites), and internal control Renilla luciferase reporter constructs for 24 h, then treated with SNS-032 for another 24 h, followed by luciferase activity assay. **f** SNS-032 reduced mRNA levels of YAP target genes. The UM cells were treated with SNS-032 for 24 h, qRT-PCR analysis of YAP target genes *CTGF* and *CYR61* were performed. **g** SNS-032 inhibited cell viability of UM cells but not ARPE-19 cells. The cells were incubated with increasing concentrations of SNS-032 for 72 h, cell viability was measured by MTS assay. Dose-response curves from three independent experiments are shown. Error bars represent standard deviation (SD). **h** Anchorage-independent colony growth of UM cells was dramatically inhibited by SNS-032. The UM cells pretreated with different concentrations of SNS-032 were cultured in soft agar, colony-formation ability was assessed. **i** and **j** Ectopic expression of YAP attenuated the growth inhibition effect of SNS-032. UM cells stably expressing of YAP were treated with SNS-032 (0.5 μM) for the indicated durations, expression of YAP in UM cells was examined by Western blot analysis (**i**). The cell number was counted by trypan blue exclusion assay (**j**). *, *P* < 0.05; **, *P* < 0.01; ***, *P* < 0.001, one-way ANOVA, post hoc intergroup comparisons, Tukey’s test

To assess the effects of SNS-032 on UM cells, we first detected the expression of CDK7/9 in UM cells. Western blot results showed that the levels of CDK7/9 were obviously higher in human UM cells compared with those in ARPE-19 cells (Fig. 1a). CDK7/9 is implicated in regulating the CTD phosphorylation of RNA Pol II at sites (Ser 2, 5, and 7) [33]. We next examined the phosphorylation status of RNA Pol II following exposure to SNS-032. As expected, CTD phosphorylation of RNA Pol II was inhibited by SNS-032 in a dose-dependent manner in UM cells (Fig. 1b).

Because the addiction of transcription coactivator YAP oncogene-driven by mutant Gαq/11 is required for tumorigenesis of UM [11], we next examined the effect of SNS-032 on YAP signaling. The protein levels of YAP, phospho-YAP (S127), and its downstream targets connective tissue growth factor (CTGF) and cysteine-rich angiogenic inducer 61 (CYR61) were significantly decreased in the UM cells treated with SNS-032 (Fig. 1c). Using qRT-PCR assay, we found that SNS-032 inhibited the expression of YAP at the transcriptional level (Fig. 1d). We next determined the effect of SNS-032 on YAP-dependent gene transcription in UM cells. Twenty-four hours after co-transfection with Gal4BD-TEAD4, G5-luciferase reporter (contains five Gal4-binding sites), and *Renilla* luciferase reporter constructs, Mel270 and Omm2.3 cells were treated with SNS-032 for 24 h, dual luciferase activity assay was performed. The results indicated that SNS-032 significantly reduced YAP-mediated transcriptional activities (Fig. 1e). Consistently, the mRNA levels of *CTGF* and *CYR61* genes were significantly decreased in the SNS-032-treated UM cells (Fig. 1f).

We next examined the effect of SNS-032 on cellular proliferation using MTS assay. SNS-032 significantly reduced cell viability in a concentration-dependent fashion in UM cells (IC_50_ values rang: 0.35~0.94 μM), but not in ARPE-19 cells (IC_50_ value: 20.1 μM) (Fig. 1g). Consistently, SNS-032 dose-dependently inhibited the colony-formation ability of UM cells in soft agar (IC_50_ values rang: 0.12~0.82 μM) (Fig. 1h). To examine the critical role of YAP in SNS-032-mediated growth inhibition effect in UM cells. Mel270 and Omm2.3 cells stably expressing vector or PIP-HA-YAP were exposed to SNS-032 (0.5 μM), then subjected to Western blot analysis and trypan blue exclusion assay. Ectopic expression of YAP rescued SNS-032-induced decrease in cell growth (Fig. 1i and j). Taken together, these results demonstrate that SNS-032 potently inhibits proliferation of UM cells through blocking YAP signaling.

### SNS-032 induces apoptosis in UM cells

**Fig. 2:**
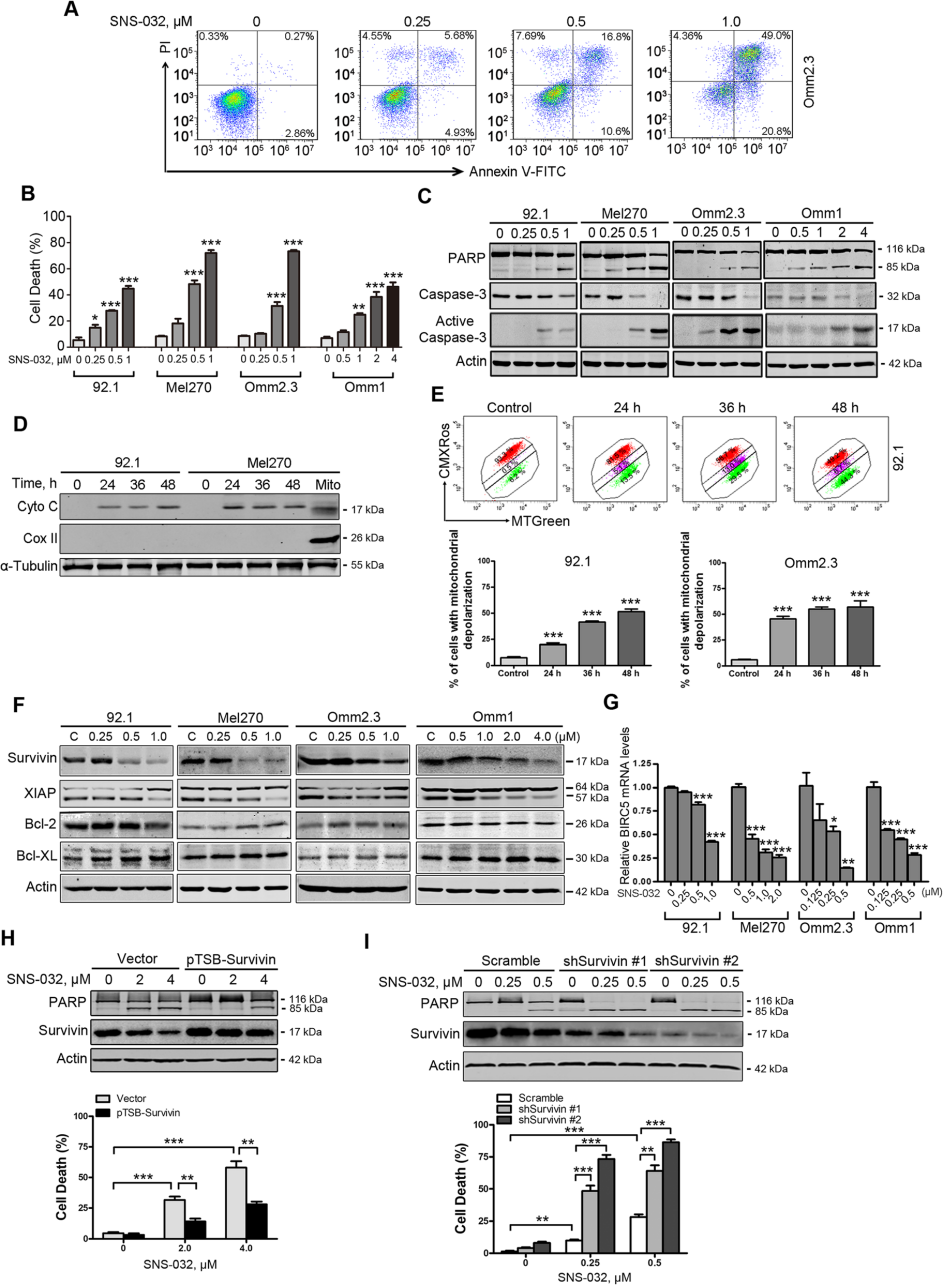
SNS-032 induces apoptosis in human UM cells. **a** and **b** UM cells were incubated with increasing concentrations of SNS-032 for 48 h, apoptosis was determined by flow cytometry after Annexin V-FITC/PI dual staining. **a** Representative flow dot plots for Omm2.3 are shown; **b** Quantitative analysis from three independent experiments. Data represent mean ± SEM. *, *P* < 0.05; **, *P* < 0.01; ***, *P* < 0.001, one-way ANOVA, post hoc intergroup comparisons, Tukey’s test. **c** Dose-dependent cleavage of PARP and activation of caspase-3 were detected by Western blot after exposure with SNS-032 for 48 h in UM cells. **d** Western blot analysis of levels of cytochrome c in the cytosolic fractionations was performed in UM cells treated with SNS-032 (1.0 μM) for the indicated durations. The cytosolic fractionations were not contaminated as indicated by a mitochondria marker COX II. **e** SNS-032 induced mitochondrial membrane depolarization in UM cells. 92.1 and Omm2.3 cells incubated with SNS-032 were stained with CMXRos and MTGreen, and then analyzed by flow cytometry. Results for three independent experiments are shown. ***, *P* < 0.001, one-way ANOVA, post hoc intergroup comparisons, Tukey’s test. **f** Western blot analysis of apoptosis-related proteins expression was performed in UM cells treated with SNS-032. **g** SNS-032 dose-dependently decreased mRNA levels of *BIRC5* gene (encoding Survivin) in UM cells. *, *P* < 0.05; **, *P* < 0.01; ***, *P* < 0.001, one-way ANOVA, post hoc intergroup comparisons, Tukey’s test. **h** and **i** Overexpression of Survivin abrogated whereas knockdown of Survivin potentiated the SNS-032-induced apoptosis. **h** Omm1 cells were transduced with lentiviral Vector (pTSB) or Survivin (pTSB-Survivin) constructs for 48 h, then treated with SNS-032 for 24 h; **i** 92.1 cells were transduced with lentiviral Vector (Scramble) or Survivin shRNAs (shSurvivin #1 and #2) constructs for 48 h, then treated with SNS-032 for 24 h. The protein levels of PARP and Survivin were detected by Western blot analysis (*top*); cell death was examined by trypan blue exclusion assay (*bottom*). **, *P* < 0.01; ***, *P* < 0.001, Student’s *t* test

We next analyzed whether SNS-032 induced apoptosis in UM cells. The UM cells were exposed to various concentrations of SNS-032 for 48 h or a fixed concentration (1.0 μM) for different durations, apoptosis was measured using flow cytometry after Annexin V-FITC/PI double staining. The results showed that SNS-032 exposure elicited an obvious apoptotic cell death in a dose- and time-dependent manner (Fig. 2a and b; Additional file 3: Figure S1A). SNS-032 treatment also resulted in concentration- and time-dependent cleavage of PARP and activation of caspase-3 (Fig. 2c and Additional file 3: Figure S1B). Additionally, the protein levels of cytochrome c in the cytosolic fractionations were dramatically elevated in UM cells treated with SNS-032 (Fig. 2d). The effect of SNS-032 on mitochondrial damage was further evaluated by the increase of cell population with loss of mitochondrial potential (ΔΨm), measured by flow cytometry after CMXRos and MTGreen double staining (Fig. 2e). These results suggest that SNS-032 induces apoptosis via the intrinsic pathway in UM cells.

### Survivin plays a critical role in SNS-032-induced apoptosis in UM cells

To explore the mechanism of SNS-032-induced apoptosis in UM cells, we next detected the expression of apoptosis-related proteins. The results showed that the levels of survivin rather than Bcl-2, and Bcl-X_L_ were decreased, while the levels of XIAP were not consistently altered among the 4 UM cell lines in response to the treatment of SNS-032 (Fig. 2f). The mRNA levels of *BIRC5* (encoding survivin) were dramatically decreased upon SNS-032 treatment (Fig. 2g). We next examined the importance of survivin in SNS-032-induced apoptosis in UM cells. Ectopic expression of survivin in Omm1 cells by lentiviral construct encoding survivin attenuated SNS-032-induced apoptosis as reflected by cleavage of PARP and dead cells with trypan blue staining (Fig. 2h). Conversely, depletion of survivin in 92.1 cells by lentiviral shRNAs potentiated the sensitivity of UM cells to SNS-032 treatment (Fig. 2i). These data suggest that survivin may play a critical role in SNS-032-induced apoptosis in UM cells.

We next examined whether there was a synergy between SNS-032 and vinblastine, the first line chemotherapy for liver metastatic UM [34]. The results showed that combinational treatment with SNS-032 and vinblastine induced enhanced cell apoptosis as reflected by increase of activated caspase-3 (Additional file 3: Figure S1C) and trypan blue staining cells (Additional file 3: Figure S1D).

### SNS-032 inhibits outgrowth of xenografted UM cells and PDX tumors in NOD-SCID mice

**Fig. 3:**
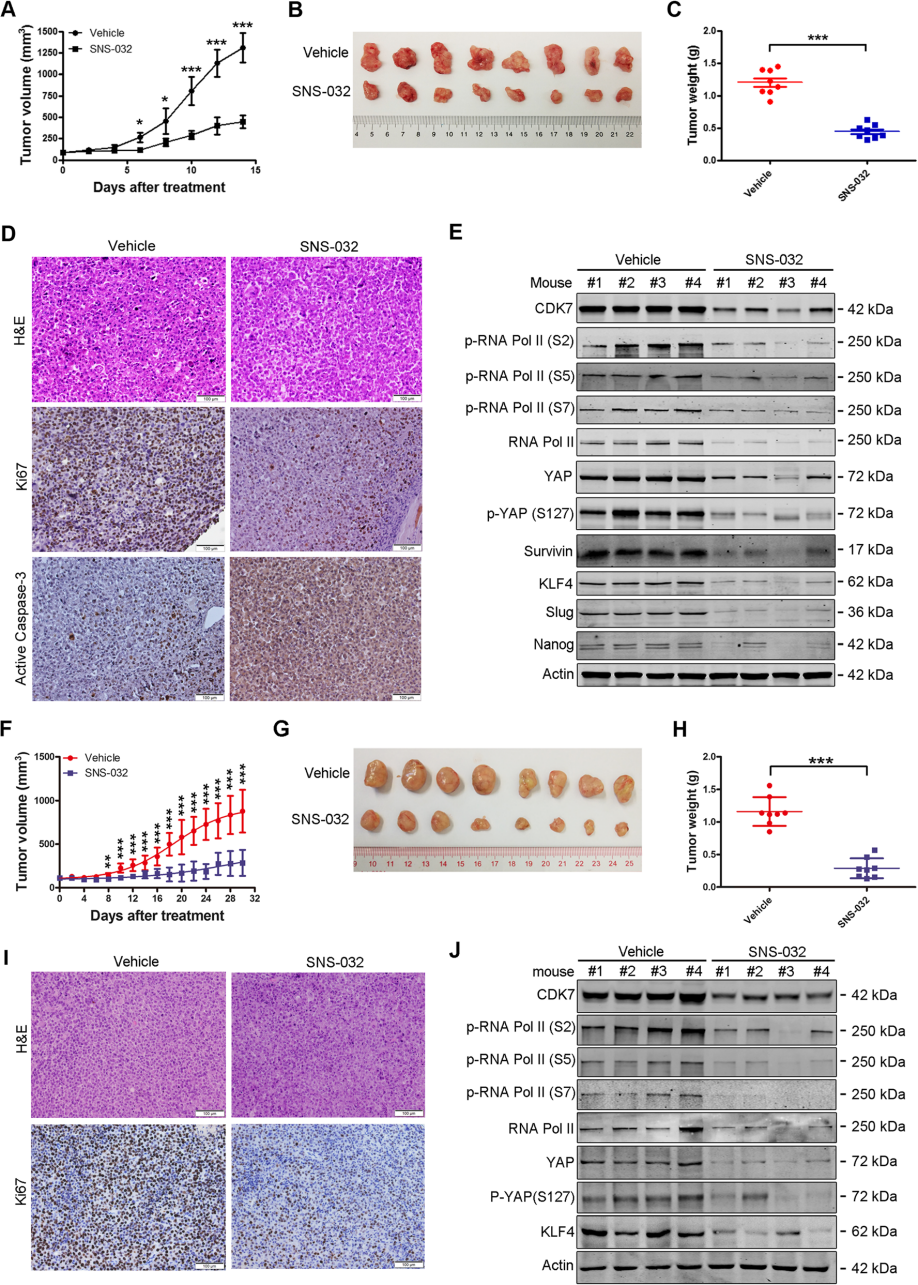
SNS-032 restrains outgrowth of xenografted UM cells and UM patient-derived xenograft (PDX) in NOD-SCID mice. **a**-**e** The effects of SNS-032 on xenografted Omm1 cells. UM xenografts were administrated with vehicle or SNS-032 for 14 days. **a** Tumor size measured every other day versus time was plotted. *, *P* < 0.05; ***, *P* < 0.001, Student’s *t* test. **b** Representative images of tumors removed from mice of each group are shown. **c** SNS-032 significantly reduced tumor weights dissected on day 15 after administration. ***, *P* < 0.001, Student’s *t* test. **d** H&E and IHC staining of tumor sections with anti-Ki67 and –active caspase-3 from mice of each group are shown. Scale bar: 100 μm. **e** SNS-032 inhibited CTD phosphorylation of RNA Pol II at Ser2, Ser5 and Ser7 sites; the expression and phosphorylation at Ser127 of YAP; as well as the expression of apoptosis-related protein Survivin and the stemness-related proteins KLF4 and Slug in tumor tissues detected by Western blot analysis with the indicated antibodies. **f**-**j** The effects of SNS-032 on UM PDX model. MP41 xenografts were administrated with vehicle or SNS-032 for 30 days. **f**-**h** The growth curves of MP41 PDX were shown, and the tumors were removed, photographed and weighed. **, *P* < 0.01; ***, *P* < 0.001, Student’s *t* test. **i** Tumor sections were performed H&E and IHC staining with anti-Ki67. Scale bar: 100 μm. **j** Cell lysates from the indicated xenografts were subjected to Western blot analysis

The in vivo antitumor effect of SNS-032 was evaluated in NOD-SCID mice bearing Omm1 xenografts subcutaneously injected. When the tumor xenografts were grown to ~ 100 mm^3^, the mice were randomly separated into two groups (*n* = 8 each) and received treatment with vehicle (PBS) or SNS-032 (15 mg/kg/day, i.p.) for 14 days. The tumor growth curve (tumor volume versus time) was significantly inhibited by SNS-032 administration (Fig. 3a). The tumor size was much smaller in SNS-032-treated group (Fig. 3b). In addition, the tumor weight was lighter in SNS-032-treated mice than vehicle-treated control ones (Fig. 3c). Furthermore, detection of proliferation marker Ki67 by IHC staining showed that the proliferation of UM cells was impaired by SNS-032 treatment (Fig. 3d). Moreover, the expression of active caspase-3 was increased in tumor tissues upon SNS-032 treatment (Fig. 3d). Cell lysates from four tumors each group were detected by Western blot analysis with the indicated antibodies. The results showed that SNS-032 inhibited the CTD phosphorylation of RNA Pol II at Ser2, 5 and 7 sites; the expression and phosphorylation at Ser127 of YAP; as well as the expression of apoptosis-related protein survivin in tumor tissues (Fig. 3e), which were consistent with the in vitro findings. No obvious side effects were observed during the treatment. Similar results were observed in MP41 PDX model which recapitulates the characteristics of human UM (Fig. 3f-j). These results demonstrate that SNS-032 inhibits the growth of UM cells in vivo.

### SNS-032 eliminates cancer stem-like cells in UM

**Fig. 4:**
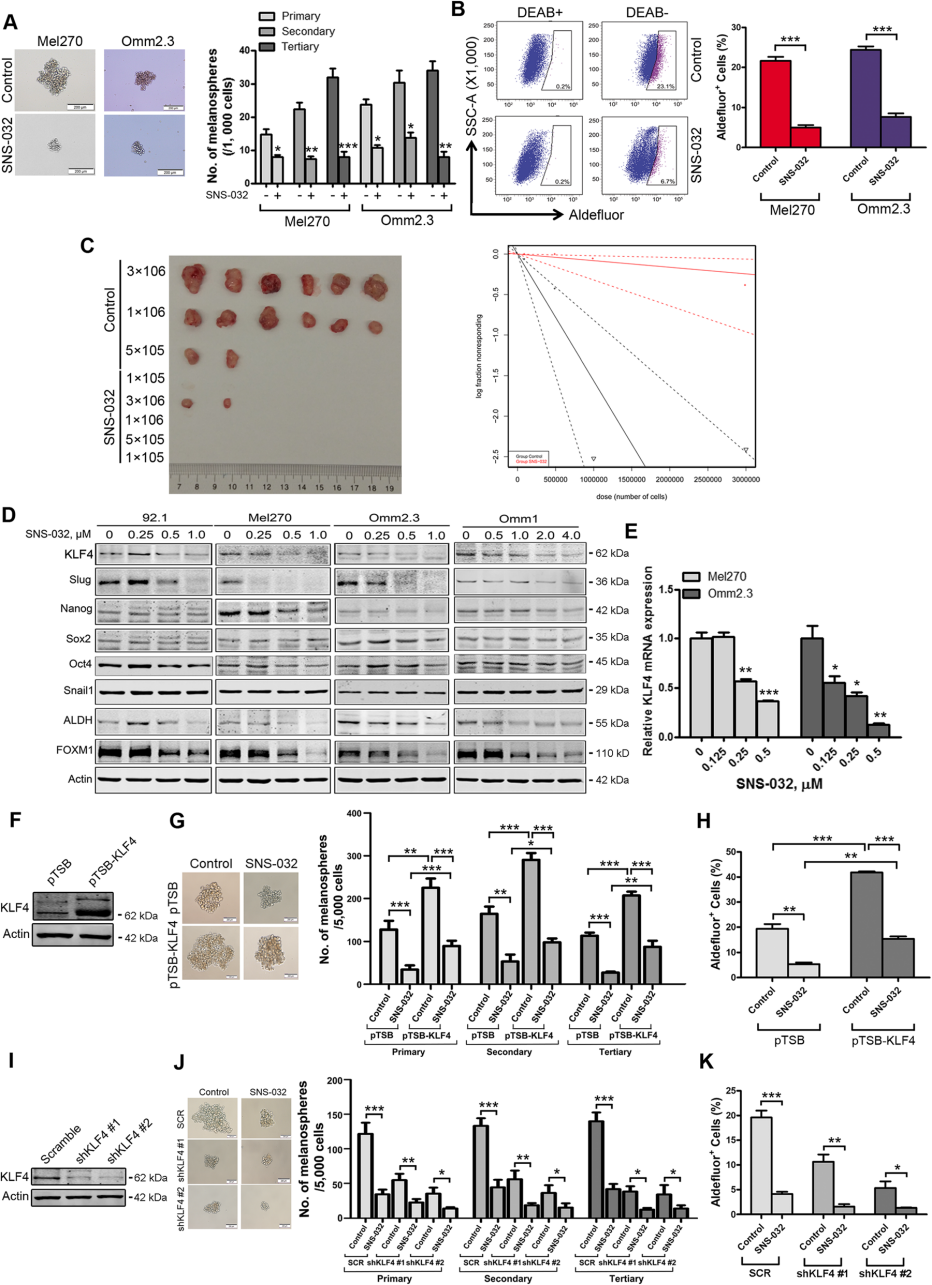
SNS-032 impairs properties of cancer stem-like cells through lowering transcription factor KLF4 in UM cells. **a** SNS-032 suppressed melanosphere growth and serially replating capacity in UM cells. Twenty-four hours after treatment with SNS-032 (0.25 μM), Mel270 and Omm2.3 cells were harvested, suspended in stem cell culture medium, and plated in ultra-low attachment 24-well plates (1000 cells/well). Melanospheres were counted on day 7. The cells were then harvested and performed for the secondary and tertiary rounds of melanosphere culture, respectively. *, *P* < 0.05; **, *P* < 0.01; ***, *P* < 0.001, Student’s *t* test. **b** SNS-032 decreased ALDH^+^ cells in UM cells. Mel270 and Omm2.3 cells were treated with SNS-032 for 48 h, the percentage of ALDH^+^ cells was detected by flow cytometry. Quantitive analysis of ALDH^+^ cells from three independent experiments is shown. ***, *P* < 0.001, Student’s *t* test. **c** SNS-032 reduced the frequency of CSCs in UM performed by limiting dilution assay in NOD-SCID mice. Representative images of tumor removed from the mice of each group are shown (*left*). The frequency of CSCs in UM is shown (*right*). **d** SNS-032 decreased the protein levels of KLF4. UM cells were exposed to SNS-032 for 48 h, the levels of stemness-related proteins were detected by Western blot analysis. **e** SNS-032 inhibited the gene transcription of *KLF4*. The mRNA levels of *KLF4* were measured after 24 h exposure to SNS-032 by qRT-PCR, and expressed as relative levels compared with controls. *, *P* < 0.05; **, *P* < 0.01; ***, *P* < 0.0001, one-way ANOVA, post hoc intergroup comparisons, Tukey’s test. **f**-**h** Ectopic expression of KLF4 attenuated SNS-032-mediated decrease of CSCs properties in UM cells. **f** The protein levels of KLF4 were examined by Western blot analysis after Mel270 cells stably transduced with lentiviral vector or construct encoding human KLF4. **g** Ectopic expression of KLF4 increases self-renewal capacity evaluated by melanosphere growth and serially replating assay. **h** Overexpression of KLF4 increases the percentage of ALDH^+^ cells detected by flow cytometry. **i**-**k** Silencing KLF4 potentiated SNS-032-mediated decrease of CSCs properties in UM cells. **i** The protein levels of KLF4 were examined by Western blot analysis after Mel270 cells stably transduced with lentiviral vector or shRNAs against human KLF4. **j** Knockdown of KLF4 decreased melanosphere growth and serially replating capacity. **k** Knockdown of KLF4 reduced the percentage of ALDH^+^ cells. *, *P* < 0.05; **, *P* < 0.01; ***, *P* < 0.001, Student’s *t* test

Given that cancer stem-like cells (CSCs) with traits such as self-renewal contribute to tumor growth, metastasis, and relapse [24], we examined the effect of SNS-032 on CSCs in UM cells. Twenty-four hours after treatment with SNS-032, the UM cells were subjected to melanosphere culture in drug-free stem cell culture medium. As illustrated in Fig. 4a, three rounds of serially replating melanosphere culture indicated that the self-renewal property of CSCs was significantly reduced by SNS-032 treatment. Elevated aldehyde dehydrogenase (ALDH) activity was a widely used biomarker for characterizing CSCs in multiple types of cancer [35]. Using the ALDEFLUOR assay followed by flow cytometry, we observed that the percentage of ALDH^+^ cells was markedly decreased upon SNS-032 treatment (Fig. 4b). We next test the effect of SNS-032 on CSCs properties in vivo by performing limiting dilution injection experiments. The results showed that SNS-032 dramatically decreased the frequency of CSCs in UM (Fig. 4c and Additional file 3: Table S3). To elucidate the underlying mechanism that SNS-032 eliminates CSCs in UM, we detected the expression of stemness-related transcription factors. SNS-032 dramatically decreased the protein levels of KLF4 and Slug (Fig. 4d). Consistently, the levels of KLF4 and Slug in tumor tissues from the mice administrated by SNS-032 were also appreciably reduced than those from the mice treated with vehicle (Fig. 3e). Analysis of The Cancer Genome Atlas (TCGA, *n* = 77) and GSE22138 database (*n* = 63) indicates that overexpression of KLF4 was strongly correlated with shorter overall survival (*P* = 0.006) and metastasis free survival (*P* = 0.014) in patients with UM, respectively (Additional file 3: Figure S2). The change of SNAI2 (encoding Slug) mRNA levels was not consistently between UM cells upon SNS-032 treatment (data not shown). Thus, we chose KLF4 for the subsequent study. The results with qRT-PCR assay showed that SNS-032 suppressed *KLF4* gene transcription in a concentration-dependent manner in UM cells (Fig. 4e). These data suggest that SNS-032 inhibits CSC properties mainly through transcription factor KLF4 in UM cells.

### KLF4 is critical for CSC elimination by SNS-032 in UM cells

To investigate the role of KLF4 in elimination of CSCs by SNS-032, we next determined the effects of manipulating KLF4 levels in UM cells. Mel270 cells stably transduced with lentiviral construct encoding human KLF4 exposed to SNS-032, the effect of ectopic expression of KLF4 on CSC properties was examined. The results showed that forced overexpression of KLF4 rescued the SNS-032-mediated decrease in capacity of serially melanosphere formation and the percentage of ALDH^+^ cells (Fig. 4f-h). In contrast, silencing KLF4 by lentiviral shRNAs enhanced the SNS-032-mediated decrease in capacity of serially melanosphere formation as well as the percentage of ALDH^+^ cells (Fig. 4i-k). Taken together, these results indicate that KLF4 plays a critical role in SNS-032-induced CSC elimination in UM cells.

### SNS-032 inhibits migration and invasion of UM cells

**Fig. 5:**
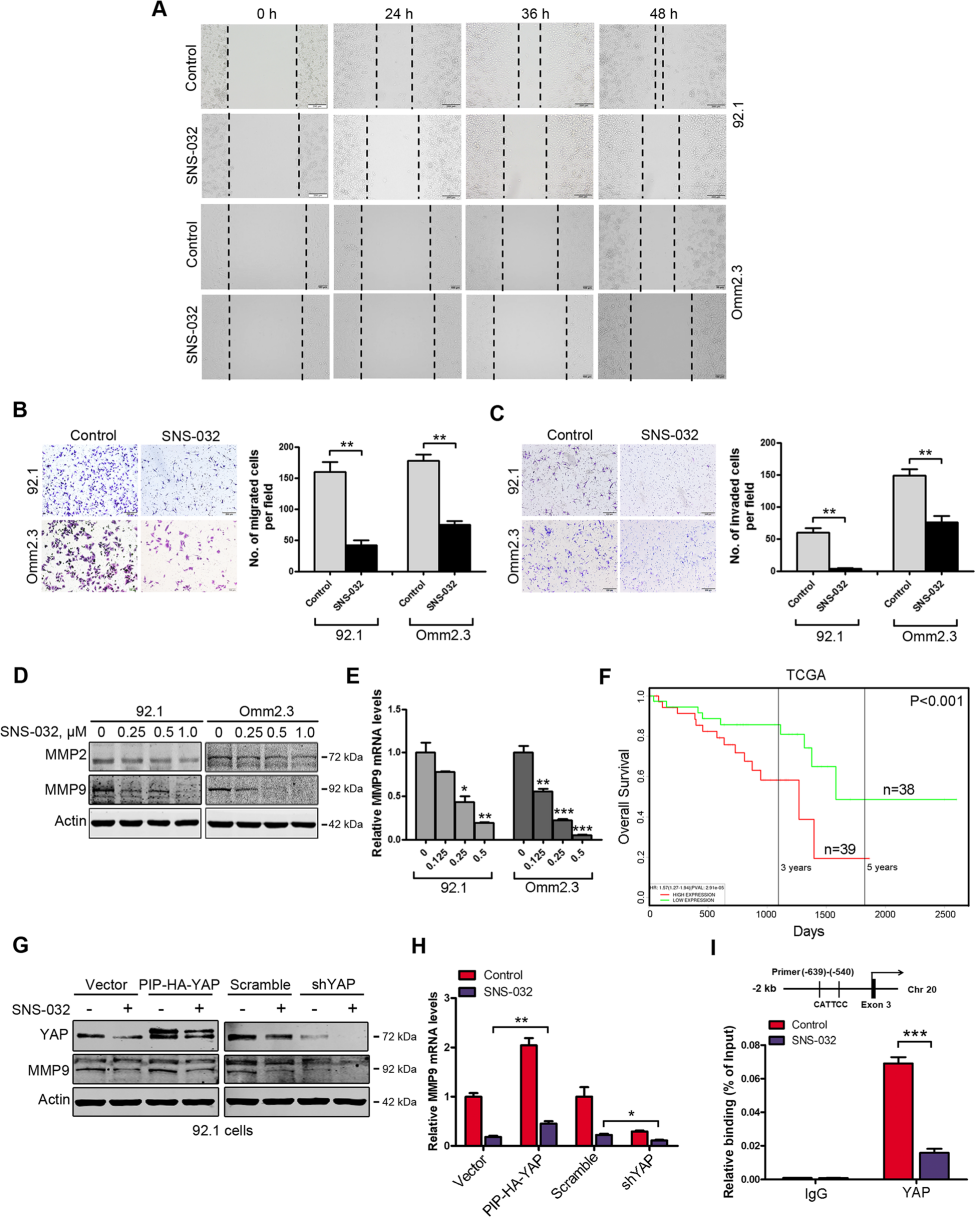
SNS-032 mitigates migration and invasion of human UM cells. **a** After treatment with SNS-032 for the indicated time periods, wound-healing scratch assay in 92.1 (*top*) and Omm2.3 (*bottom*) cells was performed. Scale bar: 200 μm. **b** SNS-032 suppressed the migration of UM cells examined by transwell assays. Migrated cells were counted in five randomly selected fields. **, *P* < 0.01, Student’s *t* test. Scale bar: 200 μm. **c** SNS-032 suppressed the invasion of UM cells examined by matrigel invasion assays. Invaded cells were counted in five randomly selected fields. Data represent mean ± SD. **, *P* < 0.01, Student’s *t* test. Scale bar: 200 μm. **d** SNS-032 inhibited the protein levels of MMP9. 92.1 and Omm2.3 cells were treated with various concentrations of SNS-032 for 48 h, MMP9 and MMP2 levels were detected by Western blot analysis. **e** SNS-032 suppressed the gene transcription of *MMP9*. The mRNA levels of *MMP9* were measured after 24 h exposure to SNS-032 by qRT-PCR. *, *P* < 0.05; **, *P* < 0.01; ***, *P* < 0.001, one-way ANOVA, post hoc intergroup comparisons, Tukey’s test. **f** Analysis of a set of UM patients (*n* = 77) from TCGA database showed that higher MMP9 expression predicted shorter overall survival (*P* < 0.001). **g** and **h** YAP was critical in SNS-032-mediated decrease of MMP9. 92.1 cells were transfected with Vector and YAP (PIP-HA-YAP) constructs (*left*) or transduced with lentiviral control (Scramble) and shRNA against YAP (*right*), then treated with SNS-032 for 24 h. Overexpression or knockdown of YAP was confirmed. The protein and mRNA levels of MMP9 were examined by Western blot and qRT-PCR analysis, respectively. *, *P* < 0.05; **, *P* < 0.01, Student’s *t* test. **i** SNS-032 reduced YAP binding to the gene promoter of *MMP9*. Mel270 cells were treated with SNS-032 (0.5 μM) for 24 h, the recruitment of YAP on the gene promoter of *MMP9* was measured by ChIP assay. IgG was used as negative control. ***, *P* < 0.001, Student’s *t* test

We investigated the effect of SNS-032 on the migration and invasion of UM cells. Wound-healing scratch assay showed that SNS-032 treatment dramatically inhibited the wound-closure capability of 92.1 and Omm2.3 cells (Fig. 5a). Similarly, the SNS-032-treated UM cells showed a significantly decreased migration ability using transwell assays (Fig. 5b). Assessed by transwell invasion assays, SNS-032 exposure dramatically decreased the number of invaded UM cells (Fig. 5c). We next examined the impact of SNS-032 on the expression of matrix metalloproteinase 9 (MMP9) and MMP2, the key metastasis-associated proteins in UM cells [36]. The levels of MMP9 but not MMP2 were decreased after SNS-032 treatment (Fig. 5d). Using qRT-PCR analysis, we found that SNS-032 inhibited the expression of MMP9 at the transcriptional level (Fig. 5e). More importantly, UM patients with higher MMP9 expression had worse prognosis from TCGA database (Fig. 5f). Because the promoter of MMP9 gene contains YAP binding sites, we hypothesized that SNS-032 may diminish the transcription of MMP9 gene via inhibiting YAP. To test it, 92.1 cells transfected with construct encoding human YAP or transduced with lentiviral shRNA against YAP exposed to SNS-032, the expression of MMP9 was examined. The results showed that the decrease of MMP9 expression by SNS-032 was partially rescued by overexpression of YAP, but enhanced by depletion of YAP assessed by Western blot and qRT-PCR analysis, respectively (Fig. 5g and h). Furthermore, treatment with SNS-032 dramatically decreased the binding of YAP to the MMP9 gene promoter examined by ChIP assay (Fig. 5i). Taken together, our data suggest that SNS-032 ameliorates migration and invasion of human UM cells through MMP9.

### SNS-032 suppresses cell motility in UM

**Fig. 6:**
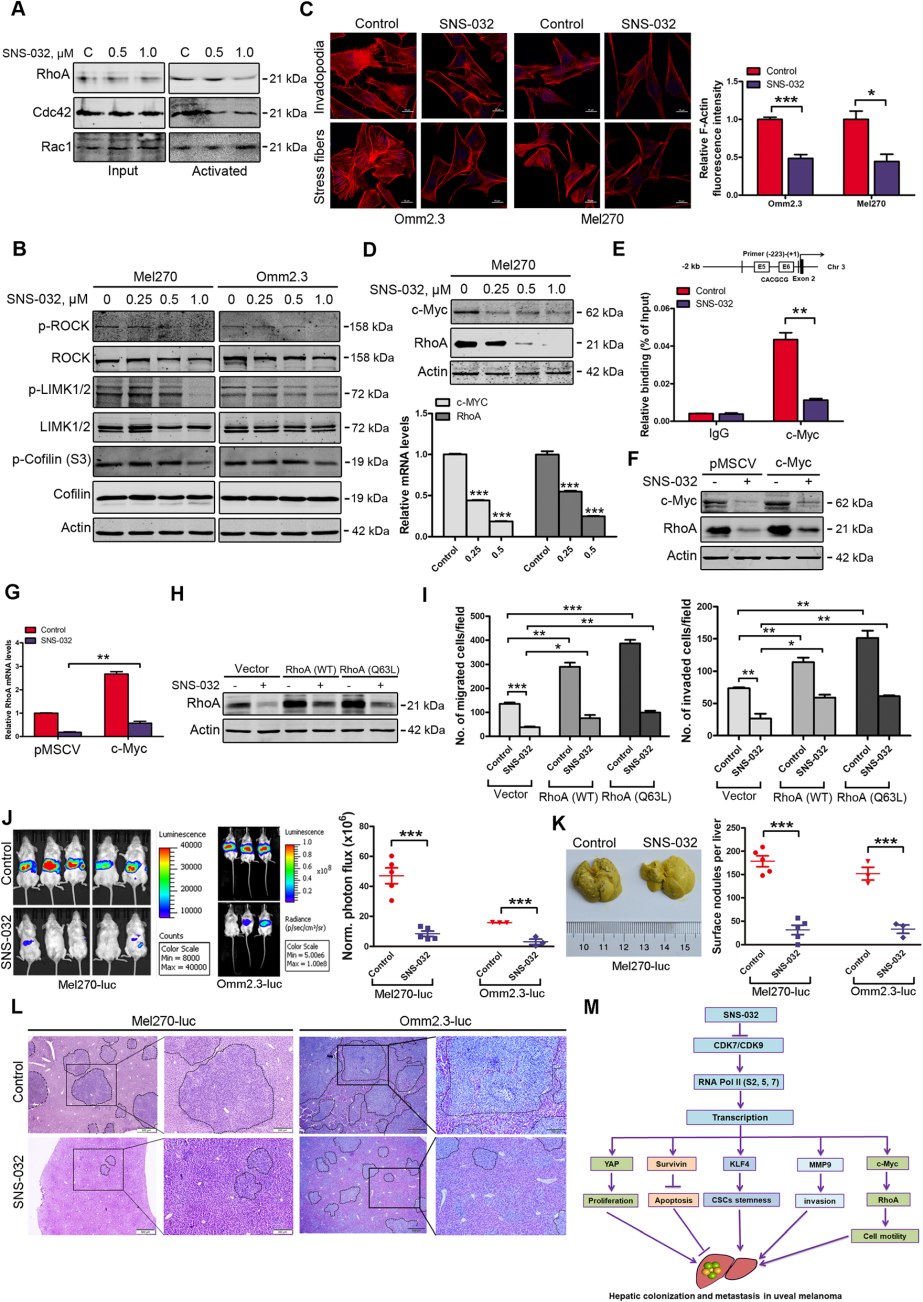
SNS-032 inhibits RhoA activity, actin polymerization, migration/invasion, and liver metastasis of UM cells. **a** SNS-032 suppressed RhoA activity. Mel270 cells were treated with SNS-032 for 24 h, active GTP-bound forms of RhoA, Cdc42 and Rac1 in cell lysates were enriched by pull-down process and detected by Western blot analysis with the indicated antibodies, respectively. **b** SNS-032 inhibited the RhoA-ROCK-LIMK1/2-cofilin pathway. Mel270 and Omm2.3 cells were treated with increasing concentrations of SNS-032 for 48 h, and then subjected to Western blot analysis with the indicated antibodies. **c** SNS-032 inhibited actin polymerization and invadopodia formation in UM cells. Twenty-four hours after treatment with SNS-032, Mel270 and Omm2.3 cells were subjected to immunofluorescence staining with phalloidin. Representative images for each group are shown (scale bar, 10 μm) (*left*); the fluorescence intensities of F-actin were quantified by Image J and normalized to control cells (*right*). *, *P* < 0.05; ***, *P* < 0.001, Student’s *t* test. **d** SNS-032 inhibited the expression of c-Myc and RhoA in UM cells. The protein and mRNA levels of c-Myc and RhoA were detected by Western blot and qRT-PCR analysis, respectively. ***, *P* < 0.001, one-way ANOVA, post hoc intergroup comparisons, Tukey’s test. **e** SNS-032 reduced c-Myc binding to the gene promoter of *RhoA* detected by ChIP assay. IgG was used as negative control. **, *P* < 0.01, Student’s *t* test. **f** and **g** Overexpression of c-Myc attenuated SNS-032-mediated decrease of RhoA. Mel270 cells transduced with retroviral c-Myc were exposed to SNS-032, the protein and mRNA levels of RhoA were assessed by Western blot and qRT-PCR analysis, respectively. **, *P* < 0.01, Student’s *t* test. **h** and **i** Overexpression of RhoA and its constitutively activated mutant (Q63L) partially rescued the SNS-032-induced decrease in capacities of migration and invasion. Mel270 cells stably expressing RhoA, RhoA (Q63L) or empty vector were exposed to SNS-032 (0.5 μM). H, the protein levels of RhoA were examined by Western blot analysis. I, the cells were underwent migration and invasion assay. **j** NOG mice were intrasplenically injected with Mel270-luc or Omm2.3-luc cells and administered with vehicle or SNS-032 (15 mg/kg/day, i.p) for 28 days, liver metastasis was measured by bioluminescent imaging (*n* = 5 per group for Mel270-luc or *n* = 3 per group for Omm2.3-luc, respectively). Representative images and quantitative analysis of photon flux on day 28 are shown. ***, *P* < 0.001, Student’s *t* test. **k** Representative images of metastatic livers for Mel270-luc cells (*left*) are shown and surface metastatic nodules in the livers are counted (*right*). Error bars represent mean ± SD. ***, *P* < 0.001, Student’s *t* test. **l** H&E-stained sections of representative livers from each group are shown. **m** A proposed working model is shown. The selective CDK7/9 inhibitor SNS-032 inhibits gene transcription by reducing the CTD phosphorylation of RNA pol II, thereby blocks YAP signaling and inhibits cellular proliferation; lowers Survivin to induce intrinsic apoptosis in UM cells. SNS-032 abolishes KLF4-dependent CSCs properties, suppresses the activation of RhoA GTPase, actin polymerization, the invasive phenotypes (e.g., migration and invasion), and ultimately diminishes liver colonization and metastasis in UM

Rho GTPase plays critical roles in regulation of cytoskeletal organization and cell motility [37]. Hence, we measured the activities of Rho GTPases (RhoA, Cdc42, and Rac1) using kinase activity assays. By pulling down the active GTP-bound form of Rho GTPases, a remarkable decrease in RhoA activity, but not Cdc42 and Rac1 was observed in SNS-032 treated Mel270 cells (Fig. 6a). Consequently, the downstream signaling pathway of RhoA was blocked (Fig. 6b) [38]. Next, we examined the effect of SNS-032 on actin polymerization. The UM cells were performed immunofluorescence analysis with Texas Red-X phalloidin staining after treatment with SNS-032. The results showed that SNS-032 significantly decreased the fluorescence signals of actin polymerization (F-actin) and the formation of invadopodia (Fig. 6c). These data demonstrate that SNS-032 decreases RhoA activity, the actin polymerization and the formation of invadopodia, and thereby inhibits the abilities of migration and invasion in UM cells.

It was reported that RhoA is implicated in cancer metastasis and its gene transcription is regulated by c-Myc [26], therefore, we detected the expression of c-Myc and RhoA after exposure to SNS-032. As respected, both the protein and mRNA levels of c-Myc and RhoA were obviously decreased (Fig. 6d). The ChIP assay indicated that SNS-032 attenuated c-Myc binding capacity to the RhoA promoter region surrounding non-canonical E-box 5 and 6 (Fig. 6e). To determine whether SNS-032 reduced the expression of RhoA through c-Myc, Mel270 cells stably transduced with c-Myc retrovirus were exposed to SNS-032, then the protein and mRNA levels of RhoA were examined. The results showed that overexpression of c-Myc partially rescued the RhoA decrease induced by SNS-032 (Fig. 6f and g).

To investigate the role of RhoA in invasive phenotypes in UM cells, Mel270 cells transfected with the constructs encoding human RhoA or a constitutively activated RhoA mutant (Q63L), then treated with SNS-032 for 24 h, transwell assay was performed. The results showed that forced overexpression of RhoA or RhoA (Q63L) promoted the migration and invasion capacities compared with the cells transfected with the empty vector. However, the decrease of migration and invasion mediated by SNS-032 was at least partially rescued by forced overexpression of RhoA or RhoA (Q63L) (Fig. 6h and i; Additional file 3: Figure S3). Taken together, these data indicate that RhoA is critical in SNS-032-induced inhibition of migration and invasion in UM cells.

### SNS-032 suppresses liver metastasis in UM

Liver metastasis is the leading cause of death in patients with UM, and no effective treatment for this fatal implication [2]. To determine the effect of SNS-032 on liver metastasis, intrasplenic injection of Mel270-luc or Omm2.3-luc cells in NOG mice was performed. Reduced bioluminescence signals in livers were observed on day 28 after SNS-032 administration (Fig. 6j). The number of metastatic nodules in surface of the livers was significantly decreased (Fig. 6k). Histologic examination indicated a remarkable decrease in number and size of the metastatic nodules in the livers in SNS-032-treated mice, compared with those in vehicle-treated mice (Fig. 6l). These results demonstrate that SNS-032 suppresses liver metastasis in UM.

## Discussions

There are no definitive therapeutic targets and no-FDA approved effective treatments for patients with metastatic UM [5]. In this study, we demonstrated that CDK7/9 is highly expressed in human UM cells, suggesting existence of elevated transcription activity. Transcription inhibition by SNS-032 significantly abrogated YAP transcription and suppressed the cellular proliferation, clonogenicity, inhibits the growth of xenografted UM cells and MP41 PDX in NOD-SCID mice. Moreover, we found that SNS-032 treatment eliminated KLF4-dependent CSCs in UM cells. SNS-032 also abrogated c-Myc addiction and inhibited c-Myc-dependent RhoA activity, actin polymerization, and consequently migration and invasion. Most importantly, SNS-032 profoundly suppressed liver metastasis in UM (Fig. 6m). Our studies provided an anti-tumor rationale of abrogating oncogene addiction by targeting transcription in UM which is a typical example with no exact metastasis-drivers identified.

It has been reported that transcriptional coactivator YAP is required for mutant Gαq/11-driven tumorigenesis in UM [10, 11]. Thus, YAP is considered as a suitable therapeutic target in UM. Our results showed that SNS-032 inhibited YAP signaling at the transcriptional level and YAP is critical in SNS-032-mediated growth inhibition in UM cells, further supporting the critical role of Gαq/11-YAP pathway in UM growth.

Sustaining proliferation and resisting cell death are hallmarks of cancer and are critical for metastasis formation and tumor growth [39]. Our results showed that SNS-032 inhibits proliferation in a panel of UM cells at nanomolar concentrations. A complete phase I clinical trial in patients with metastatic refractory solid tumors showed that the maximal plasma concentration (C_max_) of SNS-032 reached 0.75 μmol/L [21], which can be extrapolated to kill UM cells. In addition, we found that SNS-032 treatment induces intrinsic apoptosis in UM cells with survivin dramatically decreased at the transcriptional level. Consistent with our findings, it was reported that manipulation of survivin affects the efficacy of therapeutic cytotoxic-inducing agent cisplatin [40]. Becasue *BIRC5* (encoding survivin) is a well-known target gene of YAP [41], the reduced survivin may be due to the suppressed YAP by SNS-032.

Increasing evidence indicates that CSCs are believed “seeds” for tumor metastasis [42]. We found that SNS-032 treatment suppressed CSC properties in UM (the formation of melanospheres and serially replating, ALDH^+^ cells, and frequency of CSCs). It is the first report that SNS-032 eliminated CSCs in UM cells. It has been widely reported that the epithelial-to-mesenchymal transition (EMT)- and stemness-related genes including Slug, KLF4, SOX2, OCT4, and NANOG are overexpressed in human cancers and confer CSCs traits [31, 43]. The zinc-finger transcription factors KLF4 and Slug are indispensable for sustaining the CSC properties in multiple cancers, including breast, lung, and colorectal cancers [44]. We found that the change of SNAI2 (encoding Slug) mRNA levels was not consistently between UM cells upon SNS-032 treatment. Thus, Slug may not exert the critical role in SNS-032-induced CSC elimination in UM cells. The role of KLF4 in maintaining the self-renewal of CSCs in UM has not been reported. Our data showed that KLF4 is critical in the sustaining the CSC properties in UM and that CSCs is highly dependent on the transcription of stemness oncogene KLF4. Consistent with our findings, Li, et.al., reported that statins dramatically reduced CSC properties and metastasis in osteosarcoma by downregulating KLF4 [43].

We have recently reported that Slug is fundamental in sustaining the stemness of UM cells. However, Slug appeared to be largely regulated at layer of protein stability in UM. A decreased Slug protein was observed in UM cells treated by neddylation modification inhibitor MLN4924, which was at least partially explained by induction in E3 ligase FBXO11 of Slug protein [23].

Metastasis is referred to as the spread of cancer cells from primary tumor sites to distant organs [45]. Local invasion and migration of cancer cells into tissues is the early step in the metastatic cascade. MMP9, an important member of matrix metalloproteinases, is able to break down the extracellular matrix, and promote local invasion [45]. Accumulating evidence indicates that MMP9 is critical for migration and invasion of cancerous cells including triple-negative breast cancer and colorectal cancer [46, 47]. It was reported that MMP9 was mainly expressed in epithelioid UM or the epithelioid portion of mixed UM, which was associated with a higher metastatic rate [48]. MMP9 was demonstrated to be pivotal for invasion of UM cells [28]. In the present investigation, our results reveal MMP9 in UM cells are sensitive to transcription blocking.

Cytoskeletal reprogramming is a fundamental event throughout the complicated process of metastasis. The small Rho GTPases including RhoA, Cdc42, and Rac1 control the actin cytoskeletal organization and are required for cell migration [37]. It was previously reported that RhoA GTPase is implicated in cancer metastasis [26]. It is of interest to elucidate what oncogene(s) contribute cell motility of UM cells. In our study, we found that SNS-032 inhibited the activated form of RhoA and its downstream signaling, subsequently decreased actin polymerization and the formation of invadopodia. Ectopic expression of RhoA promoted migration and invasion, and partially rescued the effect of SNS-032 in UM cells. Previously report demonstrated that Gαq/11 interacts with Trio (guanine nucleotide exchange factor) to activate GTPases RhoA and Rac1, resulting in actin polymerization and F-actin accumulation [10]. Our results showed that SNS-032 has no effect on the expression of Gαq/11 (data not shown), suggesting that SNS-032 inhibits RhoA activation and actin polymerization may not through Gαq/11. The transcription factor c-Myc contributes to the tumorigenesis and metastasis. Our ChIP results revealed that SNS-032 treatment decreased binding of endogenous c-Myc to the non-canonical E-box 5 and 6 to inhibit *RhoA* gene transcription, which was consistent with the previous reports [26, 49]. In this sense, we identified c-Myc as a driver oncogene in RhoA-dependent UM cell motility by using the chemical probe of SNS-032.

Although SNS-032 was originally designed as a selective inhibitor against CDK7, CDK9 and CDK2, its inhibitory effect on CDK2 may minimally confer the antitumor activity of SNS-032 against UM cells. CDK2 and its partners cyclins A and E are drivers of S phase. However, SNS-032 did not lead to significant alternation in cell cycle distribution except a remarkable increase in sub-G1-phase cells in UM cells (data not shown). Similar results were observed in leukemia cells as described in our previous work [19]. Because blocking CDK7 and CDK9 can lead to shutdown of global transcription, and imbalance between proapoptotic and anti-apoptotic proteins, the antitumor effect of SNS-032 is likely attributed to its blockade of CDK7 and CDK9, instead of CDK2.

In conclusion, our results validate a set of transcription factors which confer metastatic traits (e.g., KLF4 for CSCs, c-Myc for cell motility) in UM cells. We discovered that CDK7/9 is highly expressed in UM cells, transcriptional inhibition by a potent and selective CDK7/9 inhibitor SNS-032 remarkably suppressed the cellular growth in vitro and in xenograft NOD-SCID mice, induced apoptotic cell death, eradicates CSCs in UM cells. Strikingly, SNS-032 dramatically inhibited the activation of RhoA GTPase and actin polymerization, and subsequently reduced the invasive phenotypes (e.g., migration and invasion) and liver metastasis in UM. Our results together identify SNS-032 as a promising therapeutic agent, and warrant a clinical trial of SNS-032 in patients with metastatic UM.

## Supplementary information

**Additional file 1: Table S1.** Primers for qRT-PCR analysis. **Table S2.** Primers for ChIP assay. **Table S3.** Limiting dilution analysis in NOD-SCID mice. (PDF 43 kb)

**Additional file 2.** Supplementary information. (PDF 77 kb)

**Additional file 3: Figure S1.** SNS-032 time-dependently induces apoptosis in human UM cells. (A) UM cells were treated with SNS-032 (1.0 μM) for the indicated incubations, cell death was examined by flow cytometry after dual staining with Annexin V-FITC/PI. Data are expressed as mean ± SEM. (B) SNS-032 induced apoptosis-specific cleavage of PARP and caspase-3 activation. UM cells were treated with SNS-032 (1.0 μM) for the indicated time periods, Western blotting analysis was performed with the specific antibodies, respectively. (C and D) MLN4924 is synergistic with vinblastine to induce apoptosis in UM cells. Mel270 and Omm1 cells were treated with SNS-032 (0.25 μM) and vinblastine (0.1 μM) for 48 h, the activation of caspase-3 was determined by Western blotting analysis (C). Cell death was detected by trypan blue staining assay. (C) *, *P*<0.05; **, *P*<0.01; ***, *P*<0.001, one-way ANOVA, *post hoc* intergroup comparisons, Tukey's test. **Figure S2.** KLF4 overexpression is strongly correlated with shorter overall and metastasis free survival in patients with UM. Analysis of The Cancer Genome Atlas (TCGA, *n*=77) and GSE22138 database (*n*=63), KLF4 overexpression was strongly correlated with shorter overall survival (*P*=0.006) and metastasis free survival (*P*=0.014) in UM patients, respectively. **Figure S3.** RhoA is critical in SNS-032-mediated decrease in migration and invasion. Mel270 cells stably expressing RhoA, RhoA (Q63L) or empty vector were exposed to SNS-032 (0.5 μM), the cells were underwent migration and invasion assay. (PDF 312 kb)

## Data Availability

Additional File 1: Supplementary Tables.pdf Additional File 2: Supplementary information.pdf Additional File 3: Supplementary Figures S1-F3 with Figure legends.pdf

## Abbreviations

ALDH: Aldehyde dehydrogenase
CDK7/9: Cyclin-dependent kinase 7/9
ChIP: Chromatin immunoprecipitation
CSCs: Cancer stem-like cells
CTGF: Connective tissue growth factor
CYR6: Cysteine-rich angiogenic inducer 61
FITC: Fluoroisothiocyanate
GNAQ/GNA11 (Gαq/11): G-protein alpha (Gα) subunits
H&E: Haematoxylin and eosin
IHC: Immunohistochemical
IVIS: In vivo imaging system
KLF4: Krüppel-like factor 4
MMP9: Matrix metalloproteinase 9
PDX: Patient-derived xenograft
PI: Propidium iodide
qRT-PCR: Real-time quantitative RT-PCR
RNA Pol II: RNA polymerase II
shRNAs: Short hairpin RNAs
TCGA: The Cancer Genome Atlas
UM: Uveal melanoma
YAP: Yes-associated protein
