# Correction to: Transcriptional inhibition by CDK7/9 inhibitor SNS-032 abrogates oncogene addiction and reduces liver metastasis in uveal melanoma

**DOI:** 10.1186/s12943-021-01410-x

**Published:** 2021-10-16

**Authors:** Jing Zhang, Shenglan Liu, Qianyun Ye, Jingxuan Pan

**Affiliations:** State Key Laboratory of Ophthalmology, Zhongshan Ophthalmic Center, Sun Yat-Sen University, 54 South Xianlie Road, Guangzhou, 510060 People’s Republic of China

**Correction to: Mol Cancer 18, 140 (2019)**

**https://doi.org/10.1186/s12943-019–1070-7**

Following publication of the original article [1], minor errors were identified in the images presented in Figs. 1, 2 and 4; specifically:

- Fig. 1b: immunoblot band for p-RNA Pol II (S2), Omm2.3 cells, has been replaced with the correct image
- Fig. 1c: immunoblot bands for p-YAP (S127), in both Mel270 and Omm2.3 cells, have been replaced with the correct images
- Fig. 2c: immunoblot band for Actin, Mel270 cells, has been replaced with the correct image
- Fig. 4d: immunoblot bands for Sox2, in both 92.1 and Mel270 cells, have been replaced with the correct images

**Fig. 1:**
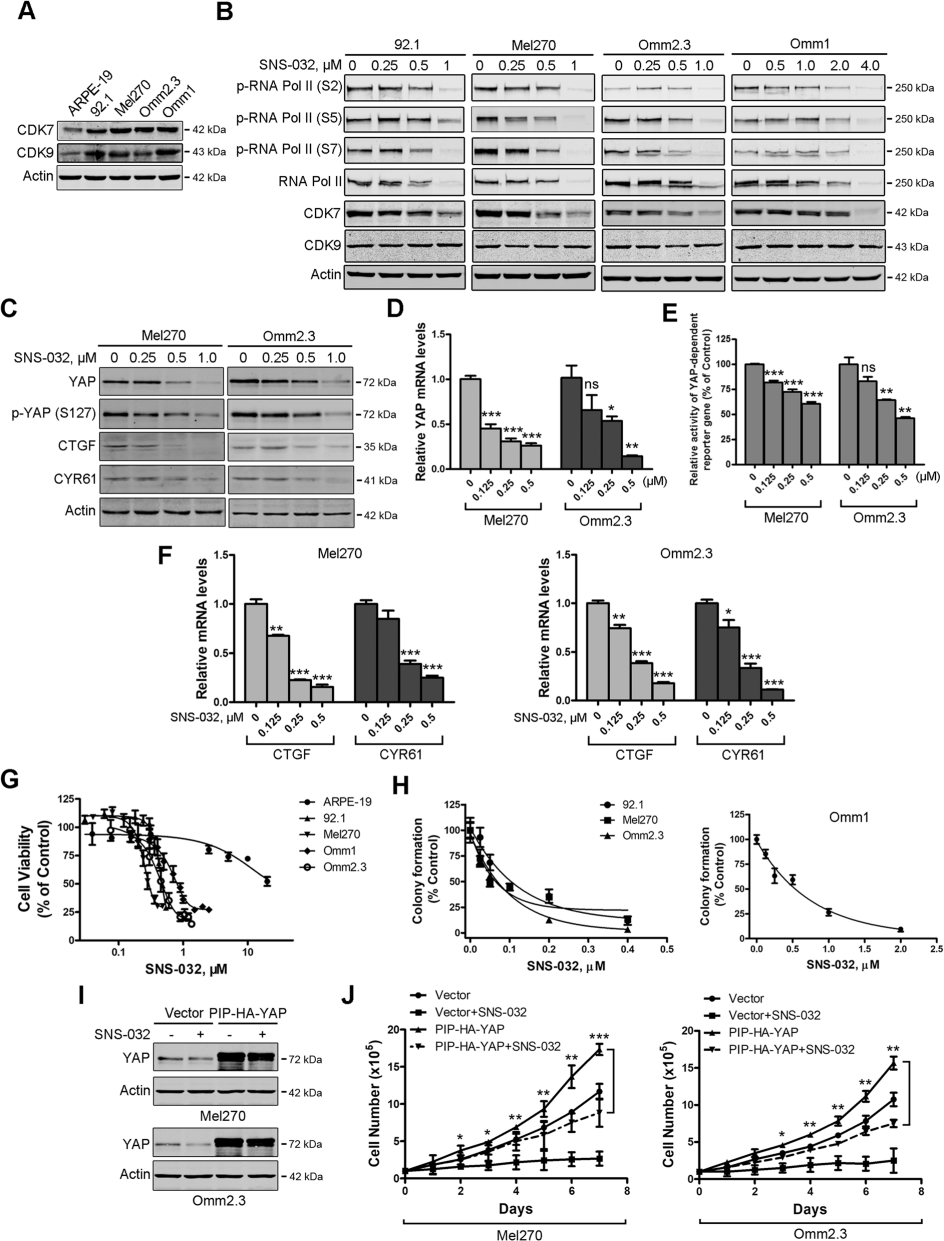
SNS-032 inhibits cellular growth through blocking YAP signaling in uveal melanoma (UM) cells. **a** The protein levels of CDK7/9 in UM cells and ARPE-19 cells were examined by Western blot analysis. **b** SNS-032 inhibited CTD phosphorylation of RNA Pol II in UM cells. Cells were treated with increasing concentration of SNS-032 for 48 h, Western blot analysis with the indicated antibodies was performed. **c** SNS-032 blocked YAP signaling in UM cells. Mel270 and Omm2.3 cells were treated with SNS-032 for 48 h, the protein levels of YAP, phospho-YAP (S127) and its downstream targets CTGF and CYR61 were examined by Western blot analysis. **d** SNS-032 inhibited gene transcription of *YAP*. The UM cells were treated with SNS-032 for 24 h, the mRNA levels of *YAP* were detected by qRT-PCR analysis. **e** SNS-032 suppressed YAP-mediated gene transcription. UM cells were co-transfected with Gal4BD-TEAD4, G5-luciferase reporter (contains five Gal4-binding sites), and internal control Renilla luciferase reporter constructs for 24 h, then treated with SNS-032 for another 24 h, followed by luciferase activity assay. **f** SNS-032 reduced mRNA levels of YAP target genes. The UM cells were treated with SNS-032 for 24 h, qRT-PCR analysis of YAP target genes *CTGF* and *CYR61* were performed. **g** SNS-032 inhibited cell viability of UM cells but not ARPE-19 cells. The cells were incubated with increasing concentrations of SNS-032 for 72 h, cell viability was measured by MTS assay. Dose–response curves from three independent experiments are shown. Error bars represent standard deviation (SD). **h** Anchorage-independent colony growth of UM cells was dramatically inhibited by SNS-032. The UM cells pretreated with different concentrations of SNS-032 were cultured in soft agar, colony-formation ability was assessed. **i** and **j** Ectopic expression of YAP attenuated the growth inhibition effect of SNS-032. UM cells stably expressing of YAP were treated with SNS-032 (0.5 μM) for the indicated durations, expression of YAP in UM cells was examined by Western blot analysis (**i**). The cell number was counted by trypan blue exclusion assay (**j**). *, *P* < 0.05; **, *P* < 0.01; ***, *P* < 0.001, one-way ANOVA, post hoc intergroup comparisons, Tukey’s test

**Fig. 2:**
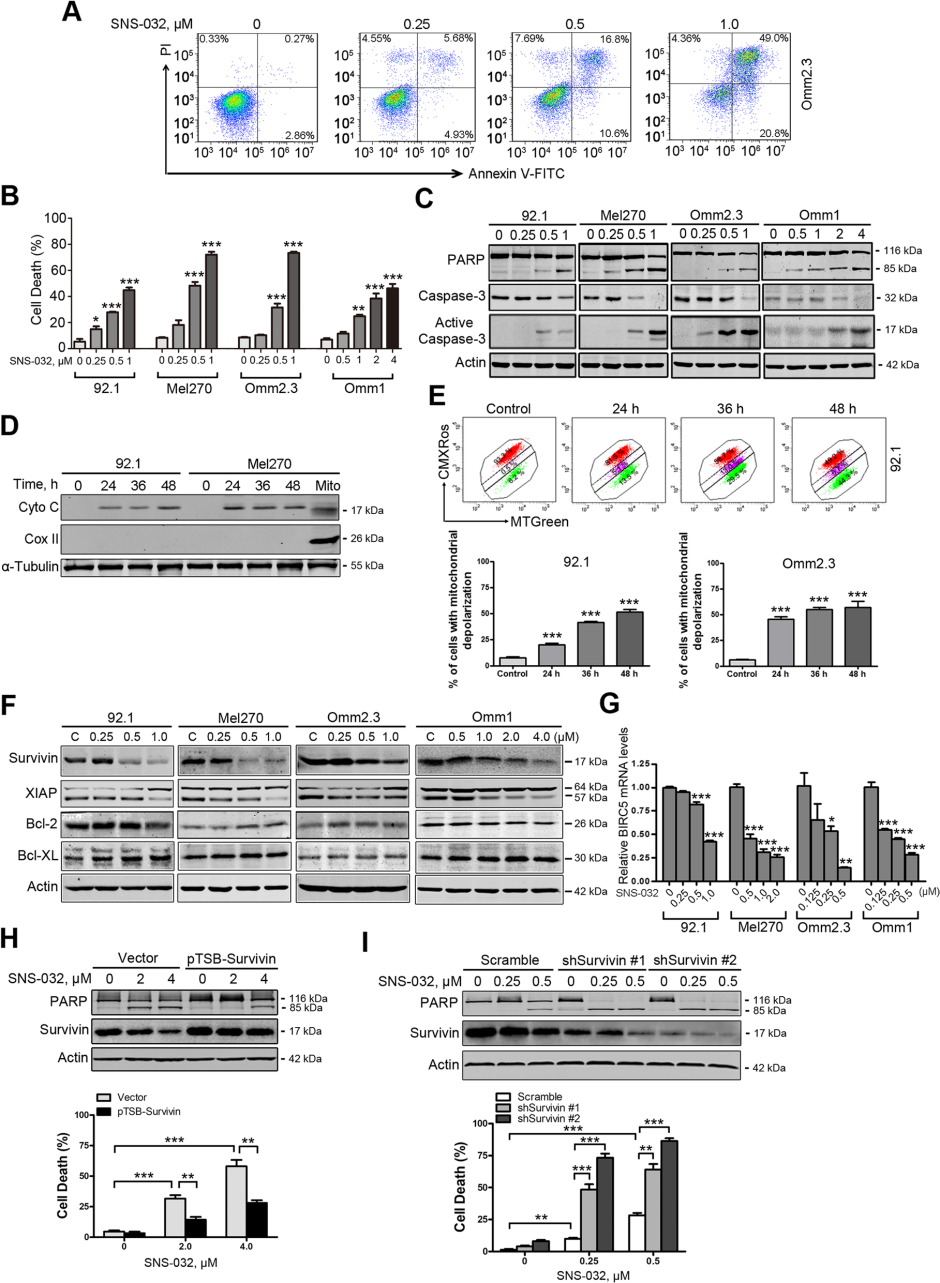
SNS-032 induces apoptosis in human UM cells. **a** and **b** UM cells were incubated with increasing concentrations of SNS-032 for 48 h, apoptosis was determined by flow cytometry after Annexin V-FITC/PI dual staining. **a** Representative flow dot plots for Omm2.3 are shown; **b** Quantitative analysis from three independent experiments. Data represent mean ± SEM. *, *P* < 0.05; **, *P* < 0.01; ***, *P* < 0.001, one-way ANOVA, post hoc intergroup comparisons, Tukey’s test. **c** Dose-dependent cleavage of PARP and activation of caspase-3 were detected by Western blot after exposure with SNS-032 for 48 h in UM cells. **d** Western blot analysis of levels of cytochrome c in the cytosolic fractionations was performed in UM cells treated with SNS-032 (1.0 μM) for the indicated durations. The cytosolic fractionations were not contaminated as indicated by a mitochondria marker COX II. **e** SNS-032 induced mitochondrial membrane depolarization in UM cells. 92.1 and Omm2.3 cells incubated with SNS-032 were stained with CMXRos and MTGreen, and then analyzed by flow cytometry. Results for three independent experiments are shown. ***, *P* < 0.001, one-way ANOVA, post hoc intergroup comparisons, Tukey’s test. **f** Western blot analysis of apoptosis-related proteins expression was performed in UM cells treated with SNS-032. **g** SNS-032 dose-dependently decreased mRNA levels of *BIRC5* gene (encoding Survivin) in UM cells. *, *P* < 0.05; **, *P* < 0.01; ***, *P* < 0.001, one-way ANOVA, post hoc intergroup comparisons, Tukey’s test. **h** and **i** Overexpression of Survivin abrogated whereas knockdown of Survivin potentiated the SNS-032-induced apoptosis. **h** Omm1 cells were transduced with lentiviral Vector (pTSB) or Survivin (pTSB-Survivin) constructs for 48 h, then treated with SNS-032 for 24 h; **i** 92.1 cells were transduced with lentiviral Vector (Scramble) or Survivin shRNAs (shSurvivin #1 and #2) constructs for 48 h, then treated with SNS-032 for 24 h. The protein levels of PARP and Survivin were detected by Western blot analysis (*top*); cell death was examined by trypan blue exclusion assay (*bottom*). **, *P* < 0.01; ***, *P* < 0.001, Student’s *t* test

**Fig. 4:**
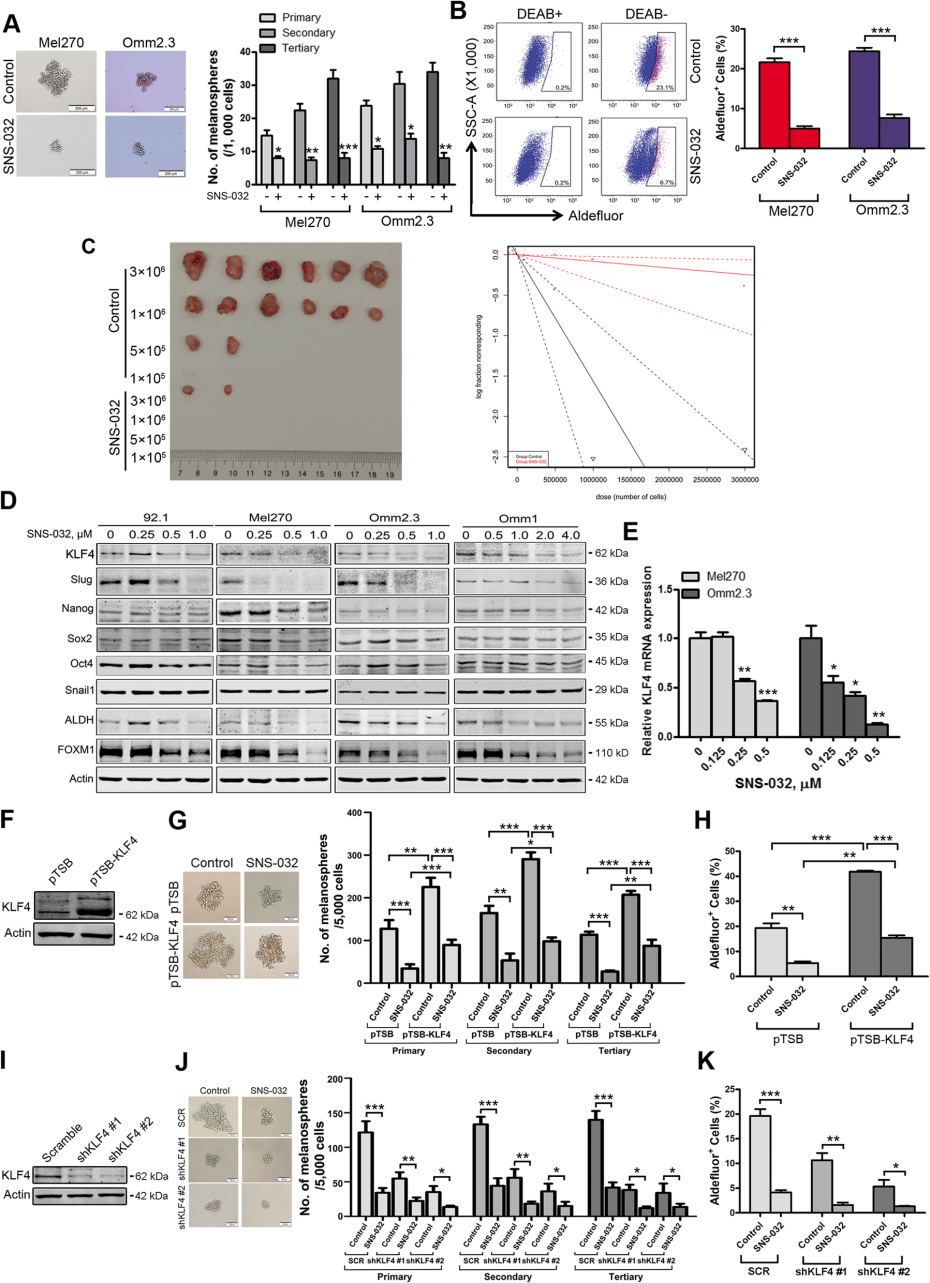
SNS-032 impairs properties of cancer stem-like cells through lowering transcription factor KLF4 in UM cells. **a** SNS-032 suppressed melanosphere growth and serially replating capacity in UM cells. Twenty-four hours after treatment with SNS-032 (0.25 μM), Mel270 and Omm2.3 cells were harvested, suspended in stem cell culture medium, and plated in ultra-low attachment 24-well plates (1000 cells/well). Melanospheres were counted on day 7. The cells were then harvested and performed for the secondary and tertiary rounds of melanosphere culture, respectively. *, *P* < 0.05; **, *P* < 0.01; ***, *P* < 0.001, Student’s *t* test. **b** SNS-032 decreased ALDH^+^ cells in UM cells. Mel270 and Omm2.3 cells were treated with SNS-032 for 48 h, the percentage of ALDH^+^ cells was detected by flow cytometry. Quantitive analysis of ALDH^+^ cells from three independent experiments is shown. ***, *P* < 0.001, Student’s *t* test. **c** SNS-032 reduced the frequency of CSCs in UM performed by limiting dilution assay in NOD-SCID mice. Representative images of tumor removed from the mice of each group are shown (*left*). The frequency of CSCs in UM is shown (*right*). **d** SNS-032 decreased the protein levels of KLF4. UM cells were exposed to SNS-032 for 48 h, the levels of stemness-related proteins were detected by Western blot analysis. **e** SNS-032 inhibited the gene transcription of *KLF4*. The mRNA levels of *KLF4* were measured after 24 h exposure to SNS-032 by qRT-PCR, and expressed as relative levels compared with controls. *, *P* < 0.05; **, *P* < 0.01; ***, *P* < 0.0001, one-way ANOVA, post hoc intergroup comparisons, Tukey’s test. **f**–**h** Ectopic expression of KLF4 attenuated SNS-032-mediated decrease of CSCs properties in UM cells. **f** The protein levels of KLF4 were examined by Western blot analysis after Mel270 cells stably transduced with lentiviral vector or construct encoding human KLF4. **g** Ectopic expression of KLF4 increases self-renewal capacity evaluated by melanosphere growth and serially replating assay. **h** Overexpression of KLF4 increases the percentage of ALDH^+^ cells detected by flow cytometry. **i**-**k** Silencing KLF4 potentiated SNS-032-mediated decrease of CSCs properties in UM cells. **i** The protein levels of KLF4 were examined by Western blot analysis after Mel270 cells stably transduced with lentiviral vector or shRNAs against human KLF4. **j** Knockdown of KLF4 decreased melanosphere growth and serially replating capacity. **k** Knockdown of KLF4 reduced the percentage of ALDH^+^ cells. *, *P* < 0.05; **, *P* < 0.01; ***, *P* < 0.001, Student’s *t* test

The corrected figures are given below. The correction does not have any effect on the results or conclusions of the paper. The original article has been corrected.
