# Supplementary material for: Transcriptional inhibition by CDK7/9 inhibitor SNS-032 abrogates oncogene addiction and reduces liver metastasis in uveal melanoma
Source: Mol Cancer. 2019 Sep 16;18:140. doi: 10.1186/s12943-019-1070-7 (PMC6745806; doi:10.1186/s12943-019-1070-7)
Supplement: Supplementary file 1 — Additional file 1: Table S1. Primers for qRT-PCR analysis. Table S2. Primers for ChIP assay. Table S3. Limiting dilution analysis in NOD-SCID mice. (PDF 43 kb) [file 12943_2019_1070_MOESM1_ESM.pdf]

Table S1. Primers for qRT-PCR analysis.

Genes	Sense primer	Antisense primer
YAP	5'- GAACTCGGCTTCAGGTCCTC-3'	5'- GTTGCTGCTGGTTGGAGTTG-3'
CTGF	5'- CCACAGAACCACCACCCT-3'	5'- CAGTTGTAATGGCAGGCACA-3'
CYR61	5'- AATGGAGCCTCGCATCCTAT-3'	5'- CGTGTGGAGATACCAGTTCCA-3'
BIRC5	5'- CATCTCTACATTCAAGAACTGG-3'	5'- GGTTAATTCTTCAAACTGCTTC-3'
KLF4	5'- CTGGGTCTTGAGGAAGTGCT-3'	5'- GGGCAGGAAGGATGGGTAAT-3'
MMP9	5'- CAAGCTGGACTCGGTCTTTG-3'	5'- CCTGTGTACACCCACACCT-3'
c-Myc	5'- CAGCGACTCTGAGGAGGAAC-3'	5'- TCGGTTGTTGCTGATCTGTC-3'
RhoA	5'- GAGCACACAAGGCGGGAG-3'	5'- CTTGCAGAGCAGCTCTCGTAG-3'
GAPDH	5'-GATCGAATTAAACCTTATCGTCGT-3'	5'-AGCAGCAGAACTTCCACTCGGT-3'

Table S2. Primers for ChIP assay.

**Primer**	**Sequence**
MMP9-promoter Sense	5'- GATTCAGCCTGCGGAAGAC-3'
MMP9-promoter Antisense	5'- TCCTCTCCCTGCTTCATCTG-3'
RhoA-promoter Sense	5'- CTTCGCGTGCGTGAAGAGTTG-3'
RhoA-promoter Antisense	5'- CATCCACTATTGCTCAGGAGC-3'

Table S3. Limiting dilution analysis in NOD-SCID mice.

Cell number	Engrafted mice	
	Control	SNS-032
3×10^6	6/6	2/6
1×10^6	6/6	0/6
5×10^5	2/6	0/6
1×10^5	0/6	0/6
Frequency	1/639,632	1/12,238,780
