# Supplementary material for: Transcriptional inhibition by CDK7/9 inhibitor SNS-032 abrogates oncogene addiction and reduces liver metastasis in uveal melanoma
Source: Mol Cancer. 2019 Sep 16;18:140. doi: 10.1186/s12943-019-1070-7 (PMC6745806; doi:10.1186/s12943-019-1070-7)
Supplement: Supplementary file 2 — Additional file 2. Supplementary information. (PDF 77 kb) [file 12943_2019_1070_MOESM2_ESM.pdf]

Supplementary Information

Supplementary materials and methods

Reagents and DNA constructs

SNS-032 and Vinblastine were purchased from Selleck (Shanghai, China), dissolved in dimethyl sulfoxide (DMSO) for stock solution at 20 mM and stored at -20°C. Texas Red-X phalloidin and DAPI were from Molecular Probes (Thermo Fisher Scientific). YAP was subcloned into pIRESpuro-2×HA expression vector (PIP-HA-YAP) as described (1). pMSCV-c-Myc, pcDNA3-EGFP-RhoA (WT), pcDNA3-EGFP-RhoA (Q63L), and Gal4-TEAD4 constructs were purchased from Addgene (Cambridge, MA). Human survivin and KLF4 were cloned into the pTSB-CMV-MCS-SBP-tRFP-F2A-PuroR lentiviral vector by Transheep (Shanghai, China) by using ClonExpress MultiS One Step Cloning kit (Vazyme, Nanjing, China). Human survivin and KLF4 specific shRNAs were obtained from Sigma-Aldrich (Shanghai, China). The pG5-Luc which contains five Gal4-binding sites upstream of the firefly luciferase gene and Renilla luciferase reporter constructs were from Promega (Shanghai, China).

Western blot analysis

Western blot analysis was performed as described (2-4). The whole cell lysates were prepared in RIPA buffer (1 × PBS, 1% NP-40, 0.5% sodium deoxycholate, and 0.1% SDS) supplemented with 1×protease inhibitor cocktail (Roche, Shanghai, China), 10 mM β-glycerophosphate, 1 mM sodium orthovanadate, 10 mM sodium

fluoride, and 1 mM phenylmetnylsulfonyl fluoride (PMSF). Cytosolic fractionations for detection cytochrome c were prepared in digitonin extraction buffer (10 mM PIPES, 0.015% digitonin, 300 mM sucrose, 100 mM NaCl, 3 mM $MgCl_2$, 5 mM EDTA, and 1 mM PMSF). Antibodies and their sources for Western blot analysis are summarized in Supplementary Information. The membranes were scanned using the Odyssey infrared imaging system (LI-COR, Lincoln, Nebraska). β-Actin or α-tubulin were used as protein loading control.

The information of antibodies for Western blotting analysis

Antibodies and their sources were as follows: anti-mouse antibodies against poly ADP-ribose polymerase (PARP, clone 4C10-5, cat no. 51-6639GR, dilution 1:4,000), X-linked inhibitor of apoptosis protein (XIAP, cat no. 610782, dilution 1:1,000), c-Myc (cat no. 51-1485GR, dilution 1:1,000), cytochrome *c* (clone 6H2.B4, cat no. 556432, dilution 1:1,000), caspase-3 (cat no. 610322, dilution 1:1,000), ALDH (cat no. 611194, dilution 1:500) were from BD Biosciences (San Jose, CA). Anti-mouse antibody against CDK7 (cat no. sc-7344, dilution 1:1000); anti-rabbit antibodies against CDK9 (cat no. sc-484, dilution 1:1000), CYR61 (cat no. sc-13100, dilution 1:500), Bcl-X_L (cat no. sc-634, dilution 1:1000), FOXM1 (cat no. sc-502, dilution 1:500); and anti-goat antibody against CTGF (cat no. sc-14939, dilution 1:500) were from Santa Cruz Biotechnology (Dallas, TX). Antibodies against YAP (cat no. 4912S, dilution 1:1000), phospho-YAP (S127) (cat no. 4911S, dilution 1:500), Active caspase-3 (cat no. 9661S, dilution 1:1000), MMP9 (cat no. 13667S; dilution, 1:500),

MMP2 (cat no. 4022S, dilution 1:500), Slug (cat no. 9585S, dilution 1:1000), KLF4 (cat no. 4038S, dilution 1:500), Nanog (cat no. 4903S, dilution 1:500), SOX2 (cat no. 3579S, dilution 1:500), Oct4 (cat no. 2750S, dilution 1:500), RhoA (cat no. 8789S, dilution 1:1000), phospho-PAK1 (T423)/PAK2 (T402) (cat no. 2601S, dilution 1:500), PAK1 (cat no. 2602S, dilution 1:1000), phospho-LIMK1 (T508)/LIMK2 (T505) (cat no. 3841S, dilution 1:500), phospho-Cofilin (S3) (cat no. 3313S, dilution 1:500), Cofilin (cat no. 5175S, dilution 1:1000) were purchased from Cell Signaling Technology (Beverly, MA). Anti-rabbit antibody against survivin (cat no. NB500-201, dilution 1:1000) was from Novus Biologicals (Littleton, CO). Anti-COXII (cat no. 3343-1, dilution 1:1000) was from Epitomics (Burlingame, CA). Anti-rabbit antibodies against phospho-RNA Pol II (S2) (cat no. A300-654A, dilution 1:1000), phospho-RNA Pol II (S5) (cat no. A304-408A, dilution 1:1000), RNA Pol II (cat no. A300-653A, dilution 1:1000) were from Bethyl Laboratories (Montgomery, TX). Antibodies against phospho-RNA Pol II (S7) (cat no. 04-1570, dilution 1:1000), Bcl-2 (cat no. 05-729, dilution 1:1000), ROCK1/2 (cat no. 07-1458, dilution 1:1000), LIMK1/2 (cat no. MAB10750, dilution 1:1000) were EMD Millipore (Billerica, MA). Anti-Snail (cat no. ab180714, dilution 1:1000) was from Abcam (Cambridge, MA). Anti-phospho-ROCK2 (S1366) (cat no. GTX122651, dilution 1:500) was from Gene Tex (Irvine, CA). Antibodies against β-actin (clone AC-15, cat no. A5441, dilution 1:5,000) and α-tubulin (clone B-5-1-2, cat no. T5168, dilution, 1:5,000) were from Sigma-Aldrich (Shanghai, China). The secondary antibodies IRDye® 680RD goat anti-mouse (cat no. 926-68070, dilution 1:10,000) and IRDye® 800CW goat

anti-rabbit (cat no. 926-32211, dilution 1:10,000) were purchased from LI-COR Biosciences (Lincoln, NE).

Cell viability assay

Cell viability was examined by MTS assay (CellTiter 96 Aqueous One Solution reagent, Promega) (2-4). Briefly, 5, 000 cells in 50 μl RPMI1640 medium seeded in 96-well plates overnight were incubated with increasing concentrations of SNS-032 for 72 h. Control cells were treated with medium containing DMSO less than 0.1%. MTS (20 μl/well) was added into the 96-well plates at the end of treatment. The optical density was read at wave length of 490 nm. IC_{50} values were determined by curve fitting of the sigmoidal dose-response curve.

Colony-formation assay

Anchorage-independent colony growth was performed by using double layer soft agar system as described (2-4). Briefly, the UM cells pretreated with SNS-032 were resuspended in complete RPMI1640 medium (400 μl) containing 0.3% agar were plated over the layer of 0.85% agar containing medium in triplicates in 24-well plates (3, 000 cells/well) and incubated at 37°C for 21 days. Colonies (≥50 cells) were accounted under an inverted phase-contrast microscope.

Apoptosis assay by flow cytometry

Apoptosis was assessed after double staining with Annexin V-

fluoroisothiocyanate (FITC)/propidium iodide (PI) and analyzed by BD LSRFortessa flow cytometry (BD Biosciences) as previously described (2-4).

Measurement of mitochondrial transmembrane potential

The UM cells were collected after exposure with SNS-032 (1.0 μM) for different durations and incubated with MitoTracker probes CMXRos and MTGreen (Thermo Fisher Scientific) at 37°C for 1 h. The inner mitochondrial transmembrane potential (ΔΨm) was examined by flow cytometry as described previously (2-4).

Real-time quantitative RT-PCR (qRT-PCR)

Total RNA was isolated with Trizol reagent (Thermo Fisher Scientific). Reverse transcription was conducted using the maxima first strand cDNA synthesis kit (Thermo Fisher Scientific). Quantitive PCR assay was carried on CFX96 Real-Time Detection System (Bio-Rad Laboratories, Hercules, CA). The primers used are list in Supplementary Table S1. The results were normalized to those of the internal control gene glyceraldehyde-3-phosphate dehydrogenase (GAPDH) (2, 3).

Dual luciferase reporter assay

UM cells were plated in 24-well plates (5×10^4/well), and then co-transfected with Gal4-TEAD4 (0.5 μg), the promoter reporter constructs pG5-Luc (0.5 μg) and Renilla-Luc (1 ng) using polyethylenimine (PEI, Polysciences, Inc., Warrington, PA). Twenty-four hours after transfection, cells were exposed to SNS-032 for 24 h. Cells

were lysed and luciferase activities were determined using the dual-luciferase assay kit (Promega) as described (2, 4).

Melanosphere-formation assay

Twenty-four hours after treatment with SNS-032, the single-cell suspensions were seeded on 24-well ultra-low attachment plates (Corning, NY). Serum-free DMEM/F12 (1:1) supplemented with B27 (1:50, Life Technologies), epidermal growth factor (EGF) (20 ng/ml, Sigma-Aldrich) and basic fibroblast growth factor (bFGF) (20 ng/ml, Sigma-Aldrich) were used as the culture medium. Fresh medium was added every two days. Melanospheres (cells ≥ 50) were counted after 7-day incubation. Then the cells were harvested, counted and re-plated (3, 000/well) for the secondary and tertiary rounds of culture, respectively. Melanospheres were counted on day 7 after each round of re-plating (2-4).

Aldehyde dehydrogenase positive cells assay

Aldehyde dehydrogenase (ALDH) positive cells were detected using the ALDEFLUOR™ kit (Stem Cell Technologies, Vancouver, BC, Canada) as described previously (2-4). Briefly, UM cells were harvested with ALDEFLUOR™ buffer (1ml), the ALDEFLUOR™ reagent (5 μl) was added and mixed, followed by transferring the mixture (0.5 ml) to a fresh tube containing ALDEFLUOR™ DEAB reagent (5 μl). After incubation at 37°C for 45 min, the samples were centrifuged and suspended in ALDEFLUOR™ assay buffer (0.4 ml), and then detected on FACS LSR Fortessa

flow cytometry. Data were analyzed using BD FACSDiva software.

Limiting dilution assay in NOD-SCID mice

Omm1 cells were exposed to control (DMSO containing medium) or SNS-032 (1.0 μM) for 24 h. Viable cells were counted using trypan blue staining, and different cell doses per group (3×10^6, 1×10^6, 5×10^5, 1×10^5) were subcutaneously inoculated into the flanks of male NOD-SCID mice (4-6-week-old). After 2 months inoculation, the mice were euthanized and tumor xenografts were immediately removed and photographed (2). The frequency of CSCs in UM was calculated by L-Calc™ limiting dilution software (STEMCELL Technologies) and summarized in Supplementary Table S3.

Wound-healing scratch assay

UM cells (92.1 and Omm2.3) were seeded in 6-well plates and grown to confluence. The cells were washed with PBS and replaced with fresh complete RPMI1640 medium after scratched with a 200 μl sterile micropipette tip. The cells were incubated with SNS-032 (0.5 μM) for the indicated time periods. The wounded area was recorded using an inverted phase-contrast microscope (2-4).

Migration and invasion assay

Cell migration and invasion assays were performed in 24-well plates with 8-μm polycarbonate sterile membrane (Corning) as described previously (2-4). Briefly, UM

cells pretreated with SNS-032 suspended in FBS-free RPMI1640 medium (200 μl) were seeded in the upper chamber (inserts) with or without Matrigel. RPMI1640 medium (600 μl) plus 20% FBS were added to lower chamber as a source of chemoattractants. After incubation for 48 h, the inserts were fixed with 4% paraformaldehyde and the cells on the lower surface were stained with 0.5% crystal violet. The cells on the upper surface were detached with a cotton swab. The results were calculated by counting three random fields of migrated and invaded cells under an inverted phase-contrast microscope.

Lentivirus transduction

Control or target lentiviral constructs together with pCMV-dR8.2 (packing construct) and the pCMV-VSVG (envelope construct) were transfected in 293T cells with PEI reagent. Viral supernatants were collected at 48 and 72 h after transfection and purified using 0.45-μm filters. UM cells (1×10^5) were transduced two rounds with lentivirus, and selected with puromycin (1 µg/ml) for 5 days (2, 4).

F-Actin staining assay

Control or SNS-032-treated cells were grown on coverslips in 6-well plates and fixed with 4% paraformaldehyde for 20 min, and then permeabilized with 1% Triton X-100 plus 0.5% NP-40 for 15 min. The cells were blocked with 5% bovine serum albumin (BSA) for 1 h at room temperature. Cells were incubated with Texas Red-X phalloidin (1:100) for 1 h at room temperature and then stained with DAPI for 1 min.

The coverslips were mounted with anti-fade reagent. Immunofluorescence staining was observed under a confocal microscope with 63× oil lens (5).

Small GTPases activities assay

Small GTPases activities were performed using the active RhoA/Cdc42/Rac1 detection kits (Cell Signaling Technology, Beverly, MA) as reported (5, 6). Briefly, control or SNS-032-treated UM cells were harvested under nondenaturing conditions. Cell lysates were used to pull down active RhoA with GST-Rhotekin-RBD (400 μg) or active Cdc42 and Rac1 with GST-PAK1-PBD (20 μg) for 1 h at 4°C with gentle rocking. Proteins in pull-down glutathione resin were eluted in reducing sample buffer and then performed Western blot analysis with anti-RhoA, -Cdc42 and -Rac1, respectively.

Chromatin immunoprecipitation assay

The chromatin immunoprecipitation (ChIP) assay was performed using the EZ ChIP kit (EMD Millipore) as described (6, 7). Briefly, 1×10^7 viable control or SNS-032-treated cells were collected and cross-linked with formaldehyde. Then the crosslinked protein/DNA was sheared into 200-600 bp in length by sonication and precleared with 60 μl of protein G agarose for 1 h. One percent of the supernatant was kept as input. The supernatant was immunoprecipitated with anti-YAP and -c-Myc or normal rabbit/mouse IgG at 4°C with rotation overnight. The enrichment of promoter binding was analyzed by qRT-PCR and normalized by input. The primers were listed

in Supplementary Table S2.

References

1. Huang JM, Nagatomo I, Suzuki E, Mizuno T, Kumagai T, Berezov A, et al. YAP modifies cancer cell sensitivity to EGFR and survivin inhibitors and is negatively regulated by the non-receptor type protein tyrosine phosphatase 14. Oncogene. 2013;32:2220-9.
2. Jin Y, Zhang P, Wang Y, Jin B, Zhou J, Zhang J, et al. Neddylation blockade diminishes hepatic metastasis by dampening cancer stem-like cells and angiogenesis in uveal melanoma. Clin Cancer Res. 2017. PMID: 29233905.
3. Wang Y, Liu M, Jin Y, Jiang S, Pan J. In vitro and in vivo anti-uveal melanoma activity of JSL-1, a novel HDAC inhibitor. Cancer Lett. 2017;400:47-60.
4. Zhou J, Jin B, Jin Y, Liu Y, Pan J. The antihelminthic drug niclosamide effectively inhibits the malignant phenotypes of uveal melanoma in vitro and in vivo. Theranostics. 2017;7:1447-62.
5. Cao J, Wang Y, Dong R, Lin G, Zhang N, Wang J, et al. Hypoxia-Induced WSB1 Promotes the Metastatic Potential of Osteosarcoma Cells. Cancer Res. 2015;75:4839-51.
6. Chan CH, Lee SW, Li CF, Wang J, Yang WL, Wu CY, et al. Deciphering the transcriptional complex critical for RhoA gene expression and cancer metastasis. Nat Cell Biol. 2010;12:457-67.
7. Jin Y, Zhou J, Xu F, Jin B, Cui L, Wang Y, et al. Targeting methyltransferase PRMT5 eliminates leukemia stem cells in chronic myelogenous leukemia. J Clin Invest. 2016;126:3961-80.
