# Supplementary material for: Transcriptional inhibition by CDK7/9 inhibitor SNS-032 abrogates oncogene addiction and reduces liver metastasis in uveal melanoma
Source: Mol Cancer. 2019 Sep 16;18:140. doi: 10.1186/s12943-019-1070-7 (PMC6745806; doi:10.1186/s12943-019-1070-7)
Supplement: Supplementary file 3 — Additional file 3: Figure S1. SNS-032 time-dependently induces apoptosis in human UM cells. (A) UM cells were treated with SNS-032 (1.0 μM) for the indicated incubations, cell death was examined by flow cytometry after dual staining with Annexin V-FITC/PI. Data are expressed as mean ± SEM. (B) SNS-032 induced apoptosis-specific cleavage of PARP and caspase-3 activation. UM cells were treated with SNS-032 (1.0 μM) for the indicated time periods, Western blotting analysis was performed with the specific antibodies, respectively. (C and D) MLN4924 is synergistic with vinblastine to induce apoptosis in UM cells. Mel270 and Omm1 cells were treated with SNS-032 (0.25 μM) and vinblastine (0.1 μM) for 48 h, the activation of caspase-3 was determined by Western blotting analysis (C). Cell death was detected by trypan blue staining assay. (C) *, P<0.05; **, P<0.01; ***, P<0.001, one-way ANOVA, post hoc intergroup comparisons, Tukey's test. Figure S2. KLF4 overexpression is strongly correlated with shorter overall and metastasis free survival in patients with UM. Analysis of The Cancer Genome Atlas (TCGA, n=77) and GSE22138 database (n=63), KLF4 overexpression was strongly correlated with shorter overall survival (P=0.006) and metastasis free survival (P=0.014) in UM patients, respectively. Figure S3. RhoA is critical in SNS-032-mediated decrease in migration and invasion. Mel270 cells stably expressing RhoA, RhoA (Q63L) or empty vector were exposed to SNS-032 (0.5 μM), the cells were underwent migration and invasion assay. (PDF 312 kb) [file 12943_2019_1070_MOESM3_ESM.pdf]

Supplementary Figure S1

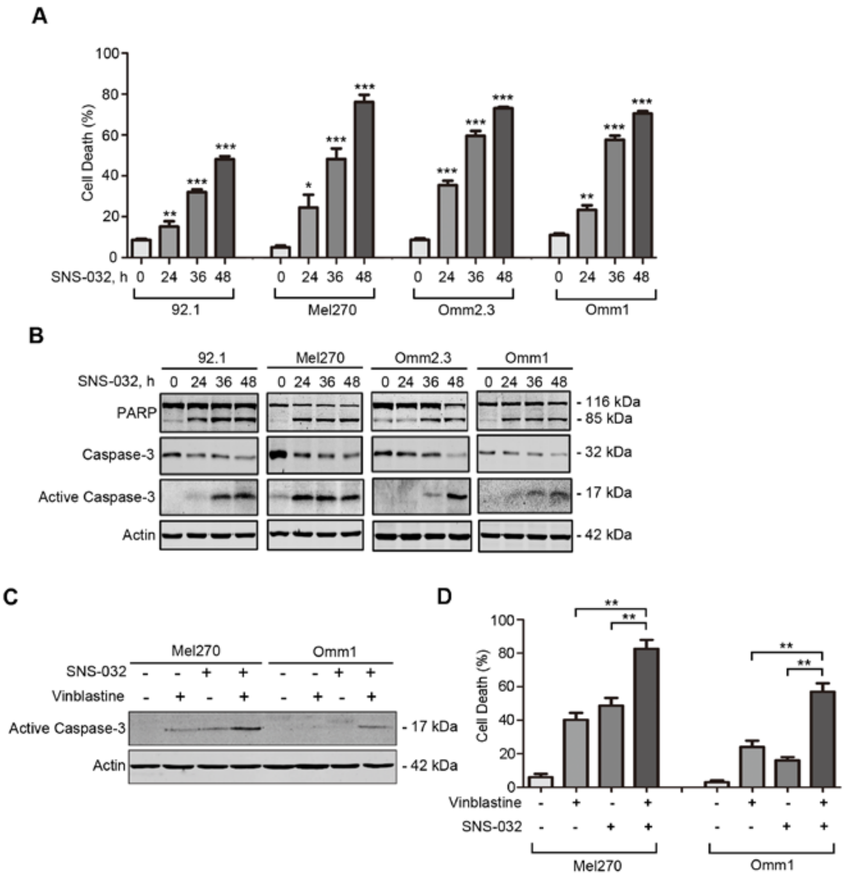

Figure S1. SNS-032 time-dependently induces apoptosis in human UM cells. (A) UM cells were treated with SNS-032 (1.0 µM) for the indicated incubations, cell death was examined by flow cytometry after dual staining with Annexin V-FITC/PI. Data are expressed as mean ± SEM. **(B)** SNS-032 induced apoptosis-specific cleavage of PARP and caspase-3 activation. UM cells were treated with SNS-032 (1.0 µM) for the indicated time periods, Western blotting analysis was performed with the specific antibodies, respectively. **(C and D)** MLN4924 is synergistic with vinblastine to induce apoptosis in UM cells. Mel270 and Omm1 cells were treated with SNS-032 (0.25 µM) and vinblastine (0.1 µM) for 48 h, the activation of caspase-3 was determined by Western blotting analysis (C). Cell death was detected by trypan blue staining assay. **(C)** *, $P<0.05$; **, $P<0.01$; ***, $P<0.001$, one-way ANOVA, *post hoc* intergroup comparisons, Tukey's test.

Supplementary Figure S2

A

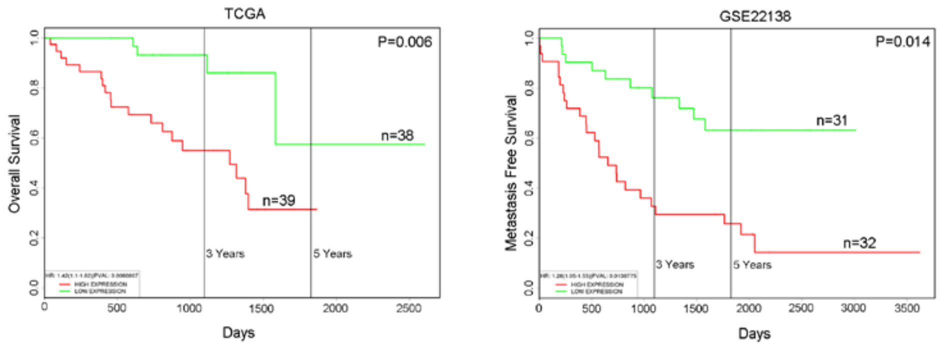

Figure S2. KLF4 overexpression is strongly correlated with shorter overall and metastasis free survival in patients with UM. Analysis of The Cancer Genome Atlas (TCGA, n=77) and GSE22138 database (n=63), KLF4 overexpression was strongly correlated with shorter overall survival (*P*=0.006) and metastasis free survival (*P*=0.014) in UM patients, respectively.

Supplementary Figure S3

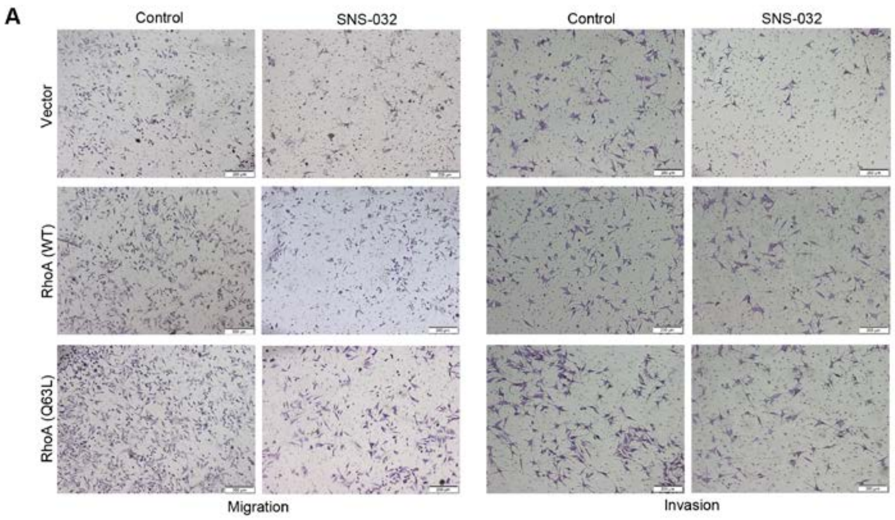

Figure S3. RhoA is critical in SNS-032-mediated decrease in migration and invasion. Mel270 cells stably expressing RhoA, RhoA (Q63L) or empty vector were exposed to SNS-032 (0.5 μM), the cells were underwent migration and invasion assay.
